# Evaluating the impact of test-trace-isolate for COVID-19 management and alternative strategies

**DOI:** 10.1371/journal.pcbi.1011423

**Published:** 2023-09-01

**Authors:** Kun Zhang, Zhichu Xia, Shudong Huang, Gui-Quan Sun, Jiancheng Lv, Marco Ajelli, Keisuke Ejima, Quan-Hui Liu

**Affiliations:** 1 College of Computer Science, Sichuan University, Chengdu, China; 2 Glasgow College, University of Electronic Science and Technology of China, Chengdu, China; 3 Department of Mathematics, North University of China, Taiyuan, China; 4 Complex Systems Research Center, Shanxi University, Taiyuan, China; 5 Laboratory for Computational Epidemiology and Public Health, Department of Epidemiology and Biostatistics, School of Public Health, Indiana University Bloomington, Bloomington, Indiana, United States of America; 6 Lee Kong Chian School of Medicine, Nanyang Technological University, Singapore, Singapore; Kirby institute, University of New South Wales, AUSTRALIA

## Abstract

There are many contrasting results concerning the effectiveness of Test-Trace-Isolate (TTI) strategies in mitigating SARS-CoV-2 spread. To shed light on this debate, we developed a novel static-temporal multiplex network characterizing both the regular (static) and random (temporal) contact patterns of individuals and a SARS-CoV-2 transmission model calibrated with historical COVID-19 epidemiological data. We estimated that the TTI strategy alone could not control the disease spread: assuming *R*_0_ = 2.5, the infection attack rate would be reduced by 24.5%. Increased test capacity and improved contact trace efficiency only slightly improved the effectiveness of the TTI. We thus investigated the effectiveness of the TTI strategy when coupled with reactive social distancing policies. Limiting contacts on the temporal contact layer would be insufficient to control an epidemic and contacts on both layers would need to be limited simultaneously. For example, the infection attack rate would be reduced by 68.1% when the reactive distancing policy disconnects 30% and 50% of contacts on static and temporal layers, respectively. Our findings highlight that, to reduce the overall transmission, it is important to limit contacts regardless of their types in addition to identifying infected individuals through contact tracing, given the substantial proportion of asymptomatic and pre-symptomatic SARS-CoV-2 transmission.

## Author summary

Among the various targeted NPIs, the Test-Trace-Isolate (TTI) strategies have been commonly adopted to mitigate and control the spread of infectious diseases. Previous studies evaluating the effectiveness of TTI strategy on SARS-CoV-2 spreading have presented contrasting results, which could be due to the nature of observation studies and imperfect reflection of characteristics of the disease and interventions including TTI in the analyses. To properly account for the contact patterns, here we propose a novel static-temporal multiplex network model describing the static contacts and temporal contacts encountered in different settings (like households, schools, workplaces or general community), which captures the essential nature of social behavior relevant to transmission risk and enables us to assess the effectiveness of the TTI strategy. Our proposed model suggests that TTI strategy solely could not control the spread of SARS-CoV-2 and it should be combined with other social distancing policy to reduce the overall transmission of SARS-CoV-2.

## Introduction

Since the first diagnosed COVID-19 case, the spread of SARS-CoV-2 has caused more than 600 million confirmed cases and 6 million deaths [1] as of 21 December 2022, which has posed overwhelming stress to the public health capacity and presenting huge challenges for the human societies [2]. To control the transmission of SARS-CoV-2 and minimize morbidity and mortality, many countries initially implemented aggressive non-pharmaceutical interventions (NPIs) such as nationwide lockdowns, at a time when effective pharmaceutical treatments were not available [3,4]. However, due to the substantial impact of the lockdowns on the functioning of the society and the availability of new options to limit COVID-19 burden such as vaccines [5], these aggressive NPIs were gradually lifted [6]. Along with vaccination campaigns, milder NPIs (e.g., masks, hand hygiene, and physical distancing) and more targeted NPIs such as reactive school-closures [7–10], screening of individuals in specific occupations [11,12], and Test-Trace-Isolate [13,14] were implemented and became crucial parts of mitigation strategies [15,16].

Among the various targeted NPIs, the Test-Trace-Isolate (TTI) strategy has been widely adopted for many infectious diseases beyond COVID-19, such as SARS [17], H7N2 influenza [18], and Ebola [19]. For COVID-19, TTI yielded contrasting results. Early studies for the UK [20], China [21], South Korea [22] and New Zealand [23] showed that a timely implementation of contact tracing has the potential to contain a SARS-CoV-2, while studies for Canada [24] and France [25] showed the opposite. These discrepancies could be explained by several reasons. First, other interventions were implemented along with TTI, which hinders the possibility to pinpoint the effectiveness of TTI. Second, TTI was implemented differently. For example, regional differences in testing capacity, tracing rate, and population compliance impact the effectiveness of TTI [26]. For example, Contreras et al. found that once the number of new cases exceeds the tracing capacity, a steep rise of new cases is expected to occur in the following weeks [26]. Hellewell et al. showed that, together with the fraction of pre-symptomatic transmission, the delay between symptom onset and isolation had the largest role in determining whether an outbreak was controllable [13]. Chiu et al.’s study indicates that it is necessary to double testing or tracing rate to contain COVID-19 in the US [27]. Davis et al. found that reporting and adherence have remarkable influence on the impact of contact tracing [28]. Third, the mitigation level of TTI is influenced by the characteristic of the disease. Indeed, effectiveness of TTI could be naturally limited by the high proportion of pre-symptomatic and asymptomatic transmission of SARS-CoV-2 [29], because the TTI is primarily triggered by identifying symptomatic patients. Oran et al. [30] estimated that nearly 40% to 45% of SARS-CoV-2 infections occur during the pre-symptomatic and asymptomatic stages, which is crucial to the effectiveness of symptom-driven interventions [31]. Furthermore, a remarkable fraction of transmission from symptomatic individuals occurs before symptom onset [32–34]. Bi et al. suggested that the effectiveness of isolation and contact tracing highly depends on the number of asymptomatic infections [21]. Thus, studies comprehensively assessing the effectiveness of TTI accounting for the characteristics of the disease and in the presence/absence of other interventions are warranted to define best practices for the use of TTI to control SARS-CoV-2 transmission.

Given that interpersonal contacts are the main transmission route for SARS-CoV-2 and other respiratory pathogens [35][36] and that TTI limits such contacts, contact patterns should be properly accounted in the assessment of the effectiveness of TTI. In fact, fomite transmission of SARS-CoV-2 is considered rare [37,38]. A key factor that limits the effectiveness of TTI is the number of contacts that an index case can remember and ultimately be traced [39]. In general, individual’s contacts can be classified into two groups [40–42]. The first group includes individuals that are met regularly, such as household members, schoolmates, and colleagues; here referred to as “static contacts” [43,44]. The second group includes individuals that are met occasionally and/or randomly [45,46], such as individuals met at the supermarket, on the bus, etc. These contacts, here referred to as “temporal contacts”, are hard to trace but were proven to be a relevant source of exposure for SARS-CoV-2 [47,48]. Despite their importance, most modeling studies that focused on accessing the impact of TTI on the spread of SARS-CoV-2 have not or only partially reflected such static-temporal structure of the contact network. For example, some studies have not considered individuals’ contact networks, and assumed homogeneous mixing either implicitly or explicitly [13,14,26,28,49]. Other studies considered complex contact patterns but connections were static [7,50]. Several studies have attempted to incorporate temporal information in the network structure [51]. One strategy that has been adopted is the use of weighted links to indicate the frequencies or duration of contacts. For example, Firth et al. [52] constructed a static social network based on social contact data and movement data and weighted each contact by the frequency of days that a contact was observed; however, it is worth noting that this type of simplification may limit the capacity of the network model to represent various distributions of epidemic sizes [53]. A second strategy was the use of line graphs to represent the movement from source to target and the corresponding time points. This method requires specific location information and chronological order, which is highly demanding for data acquisition. Because the significance of temporal contact network on pathogen transmission [47,48] and on the different effectiveness of TTI for static (relatively easy to identify) vs. temporal (difficult to identify) contacts, considering both static and temporal contact patterns into a single modeling framework will help us better evaluate effectiveness of TTI.

In this work, we developed a novel infection transmission model considering a static-temporal multiplex network. The transmission model was used to access the effectiveness of TTI on SARS-CoV-2 spread considering an epidemiological context consistent with the initial phase of the COVID-19 pandemic (i.e., ancestral SARS-CoV-2 lineages, fully susceptible population, no vaccines and antiviral therapies). We run epidemic simulations over one year for different values of the reproduction number and under different TTI strategies coupled with interventions restricting contacts. Simulated scenarios are compared in terms of COVID-19 burden (e.g., number of averted infections and deaths) and social and implementation costs of TTI (e.g., peak number of simultaneously isolated individuals, peak number of individuals that needs to be traced).

## Methods

### Overview of transmission simulation with TTI

To simulate SARS-CoV-2 transmission dynamics in a population, we developed a stochastic agent-based model [54] on a static-temporal multiplex network. The disease progression was modelled through a susceptible-exposed-infectious-removed (SEIR) scheme. The static-temporal multiplex network was composed of two layers: the static contact layer and the temporal contact layer. The links on the static contact layer describe regular contacts, such as contacts among household members, schoolmates, and colleagues, which is assumed to be static over the epidemic period. The links on the temporal contact layer describe occasional contacts, which are assumed to change over time and are updated every day in our simulation. Furthermore, we implemented TTI in the simulation and examined its effectiveness in reducing disease burdens over one year.

### Contact survey data

To reconstruct the network of contacts, we relied on data collected in a contact survey conducted between December 2017 and May 2018 in Shanghai, China [46]. Similar to the POLYMOD study [55], the survey collected basic socio-demographic information about each study participant (e.g., age, sex) and detailed information about each individual contacted by the participant during the 24 hours period before filling in the questionnaire, where a contact was defined as either a two-way conversation with three or more words in physical presence or a physical skin-to-skin contact. Information collected for each contact included age/age group, sex, contact location and duration, whether the contact was physical or not, and the social setting where the contact took place (e.g., school, workplace). The original contact survey data shows that households, schools, and workplaces were the social settings accounting for the majority of contacts but participants also reported large number of contacts (20+) that were difficult to record individually, like those related to social events (i.e., contacts in the general community).

### Static-temporal multiplex network model

**Fig 1:**
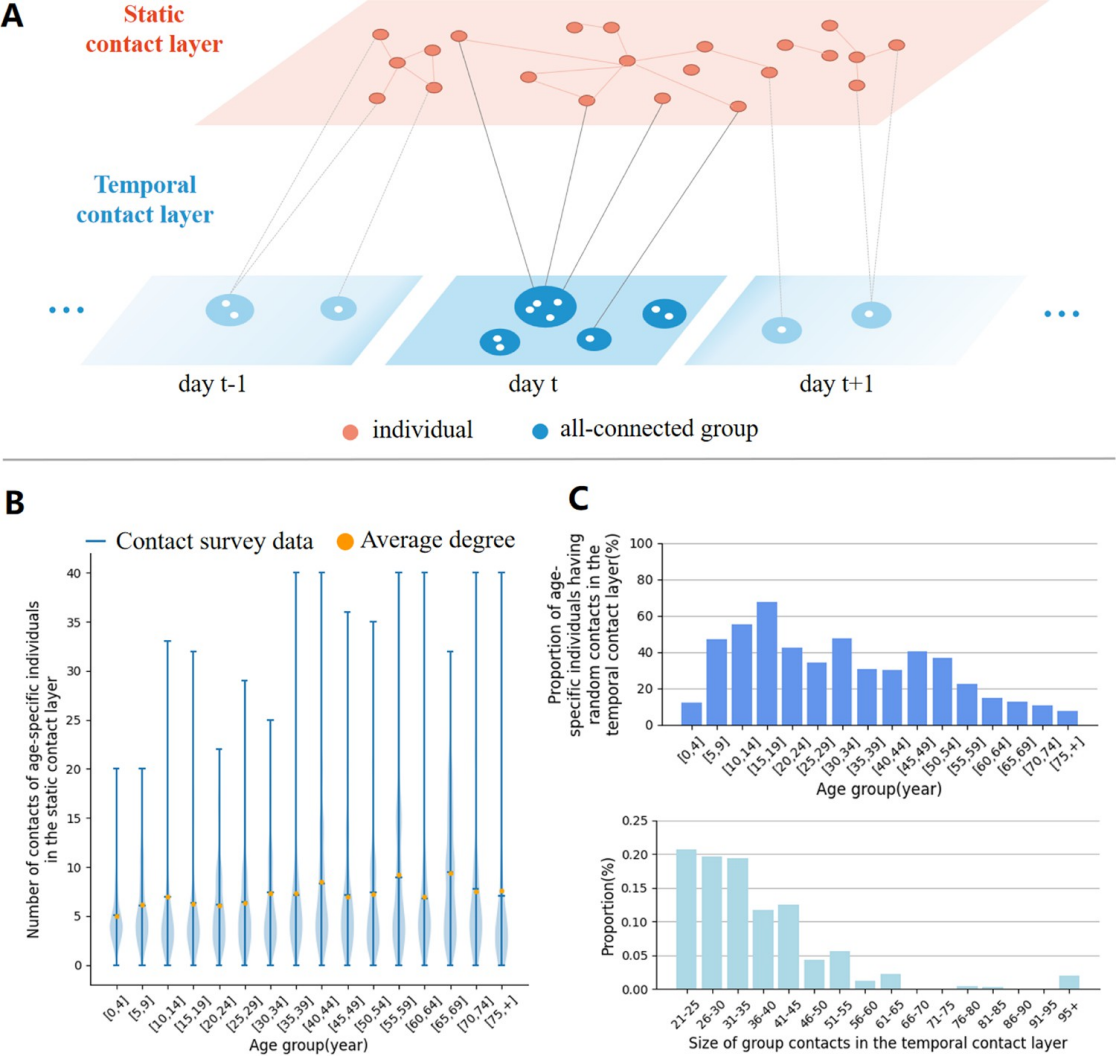
Static-temporal multiplex network model and social contact pattern. **A** A schematic illustration of the static-temporal multiplex network, which is composed of two layers: static contact layer and temporal contact layer. The links on the static contact layer represent regular interactions occurring in households, schools, and workplaces, whereas those on the temporal contact layer represent occasional contacts occur in gatherings and events. The links on the static contact layer are constant, whereas the links on the temporal layer are updated every day. **B** The age-specific contact frequency from the survey and the synthesized static layer. **C** The age-specific proportion of individuals joining gathering and events. **D** The size distribution of gathering and events.

We developed a novel static-temporal multiplex network (**Fig 1A**). The network is composed of two layers: a static contact layer and a temporal contact layer, which represent age-specific “regular” contact patterns (i.e., contacts which occur in households, schools, and workplaces) and “occasional” contact patterns (e.g., contacts taking place during social events), respectively. In each layer, nodes and links represent individuals and contacts, respectively. A population of 100,000 individuals is synthesized following the age distribution from the census data of Shanghai, China [56] and connected on the static contact layer and the temporal contact layer. A configuration model was used to construct both the static and temporal contact layers [57], preserving the age-specific regular and occasional contact patterns observed in the contact survey conducted in Shanghai, China [46]. On the static contact layer, links are generated following age-dependent contact frequencies (**Fig 1B**). Specifically, for each node, the number of links was sampled from the corresponding age-specific distribution. The static contact layer is assumed constant (thus the links are not re-generated or disappear during the simulation of an epidemic). On the temporal contact layer, social events are generated, in which all participants are connected. Social events are generated following the proportion of individuals participating to social events as derived from the contact survey data (**Fig 1C**), the distribution of the event size (**Fig 1D**), and age distributions of the participants of the social event (**S3 and S4 Figs**). Notably, school-age or working-age individuals are more likely to have contacts on the temporal contact layer (**Fig 1C**). The links on the temporal contact layer are updated at each time step of the simulation (i.e., every day). The average numbers of contacts for the static and temporal contact layers are 7.15 and 13.7, respectively, which add up to 20.85 contacts per day and is consistent with the original data (an average of 19.3 contacts per day) and other estimates obtained before the COVID-19 pandemic in European countries (e.g., 19.77 and 17.46 contacts per day on average were reported in Italy and Luxembourg, respectively) [55]. More details are available in **S1 Supplementary Methods** (1. Contact survey data for the static-temporal multiplex network and 2. Static-Temporal Multiplex Network).

### SARS-CoV-2 transmission model

We developed a stochastic agent-based model of SARS-CoV-2 transmission to evaluate the impact of the test-trace-isolate strategy in mitigating COVID-19 burden. Briefly, SARS-CoV-2 transmission is simulated according to an SLIR (susceptible, latent, infectious, removed) scheme, where infectious individuals were further divided into pre-symptomatic (P), symptomatic (S), and asymptomatic (A) individuals (**S9 Fig**). If a susceptible individual i is connected with an infectious (either pre-symptomatic, symptomatic, or asymptomatic) individual j, susceptible individual i could be infected by infectious individual j with a layer-specific probability and become latent. After a latent period, latent individuals either become infectiousness pre-symptomatic or asymptomatic. Once they develop symptoms, pre-symptomatic individuals are categorized as symptomatic. Finally, when symptomatic and asymptomatic individuals are no longer infectious, they are categorized as removed. Given that we are interested in simulating a short time period (365 days), removed individuals are assumed to be fully protected from future re-infections.

The probability of infection for each pair of infected and susceptible individuals is determined by three factors: age-specific susceptibility to infection [33], type of contact (static or temporal) [32], and the status of the infectious individual (P, I or A) [33]. The incubation period was assumed to follow a Gamma distribution with a mean of 6.3 days and a standard deviation of 4.3 (shape = 2.08, rate = 0.33) [33]. We considered transmission to start 2 days before symptom onset [33]. The duration of the infectious period was chosen such that distribution of the generation time in the simulation match an estimated mean of 7.0 days (IQR: 3.6–11.3) [32].

As a measure of disease burden, we estimated the cumulative number of infections, symptomatic infections, hospitalizations, ICUs, and deaths from the simulated epidemics. As a measure of both the social and implementation cost of TTI, we estimated the peak daily number of individuals that need to be traced (i.e., those with positive test result) and the peak daily number of individuals that are simultaneously isolated.

In our baseline analysis, we considered *R*_0_ = 2.5 (thus, if not specified, *R*_0_ = 2.5). Simulations were initialized with a fully susceptible population (thus reflecting the situation at the start of the pandemic), except for 5 randomly selected latent individuals. For each analysis, results are based on 100 stochastic model realizations. A complete list of model parameters is reported in **S1 Table**. A more detailed description of the transmission model is available in **S1 Supplementary Methods** (3. SARS-CoV-2 Transmission model).

### Test-Trace-Isolate strategy

**Fig 2:**
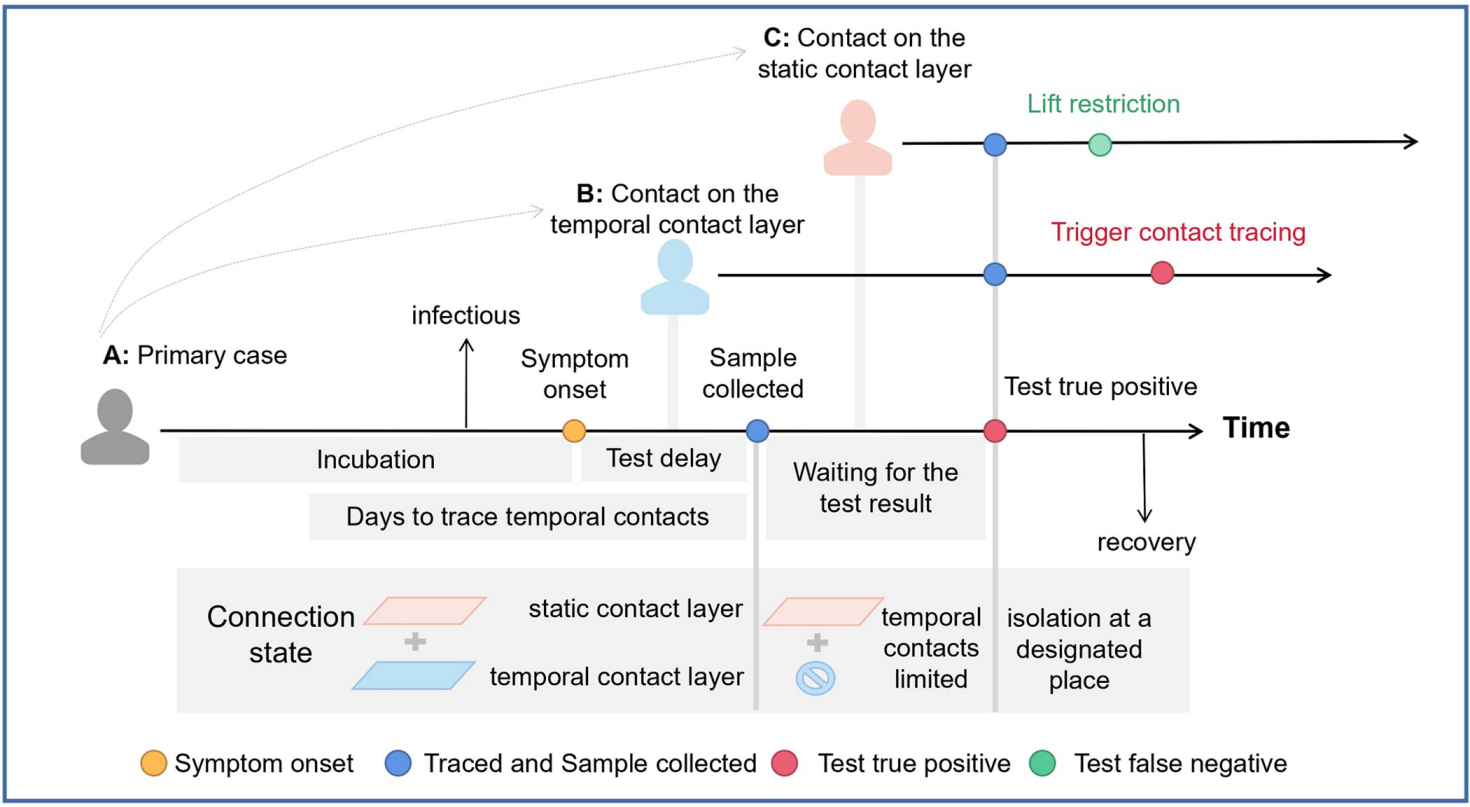
Example of the Test-Trace-Isolate strategy. Once the primary case **A** develops symptoms, the sample is collected with delay. After the sample collection, **A**’s contacts on the temporal contact layer are disconnected until the test result is returned. If the test result is positive (which is the case in this figure), **A** is isolated, thus the contacts on both layers are disconnected over 14 days. If the test result is negative (i.e., false negative), the disconnected links recover. **A** infects individual **B** through a contact on the temporal contact layer (before **A**’s sample collection) and infects individual **C** through a contact on the static contact layer before the test result is returned. Individuals **B** and **C** are traced and tested immediately after the infection of **A** is confirmed. The links of **B** and **C** on the temporal layer are disconnected until the test results are returned. If the infection of the traced individuals (**B** in this figure) is confirmed, further contact trace is triggered. If the infection of the traced individuals (**C** in this figure) is not confirmed (i.e., false negative), the disconnected links recover.

Symptom-based testing, contact tracing, and case isolation (namely, the Test-Isolate-Trace strategy) was implemented as follows:

1. **Symptom-based testing.** A fraction (80% in the baseline) of individuals who develop symptoms are tested by reverse transcription polymerase chain reaction (RT-PCR) tests. The time intervals between symptom onset and sample collection, and between sample collection and laboratory diagnosis were accounted for as well as the sensitivity of the RT-PCR test, which depends on the timing of sample collection [58].
2. **Contact tracing.** Contact tracing is triggered by a positive test result (see an example in **Fig 2**). All contacts of each positive individual on the static contact layer are traced. On the temporal contact layer, those who are connected within 4 days before sample collection are traced with a probability of 50%. The tracing process is assumed to be completed when a positive individual is identified, and the sample is immediately collected from the traced individuals.
3. **Isolation.** Before sample collection, links on both layers are active as usual. Between sample collection and test result, links on temporal contact layer are disconnected, as we assume that individuals would avoid unnecessary activities (**Fig 2**). If the test result is negative (false negative), the disconnected links recover, as individuals go back to normal activity. If the test result is positive (true positive), the links of infected individuals on both layers are disconnected (i.e., isolated) for 14 days.

Full description of the TTI strategy is available in **S1 Supplementary Methods** (5. The Test-Isolate-Trace strategy), and values and description of all TTI parameters are listed in **S2 Table**.

## Results

### Effectiveness and social/implementation cost of TTI

**Fig 3:**
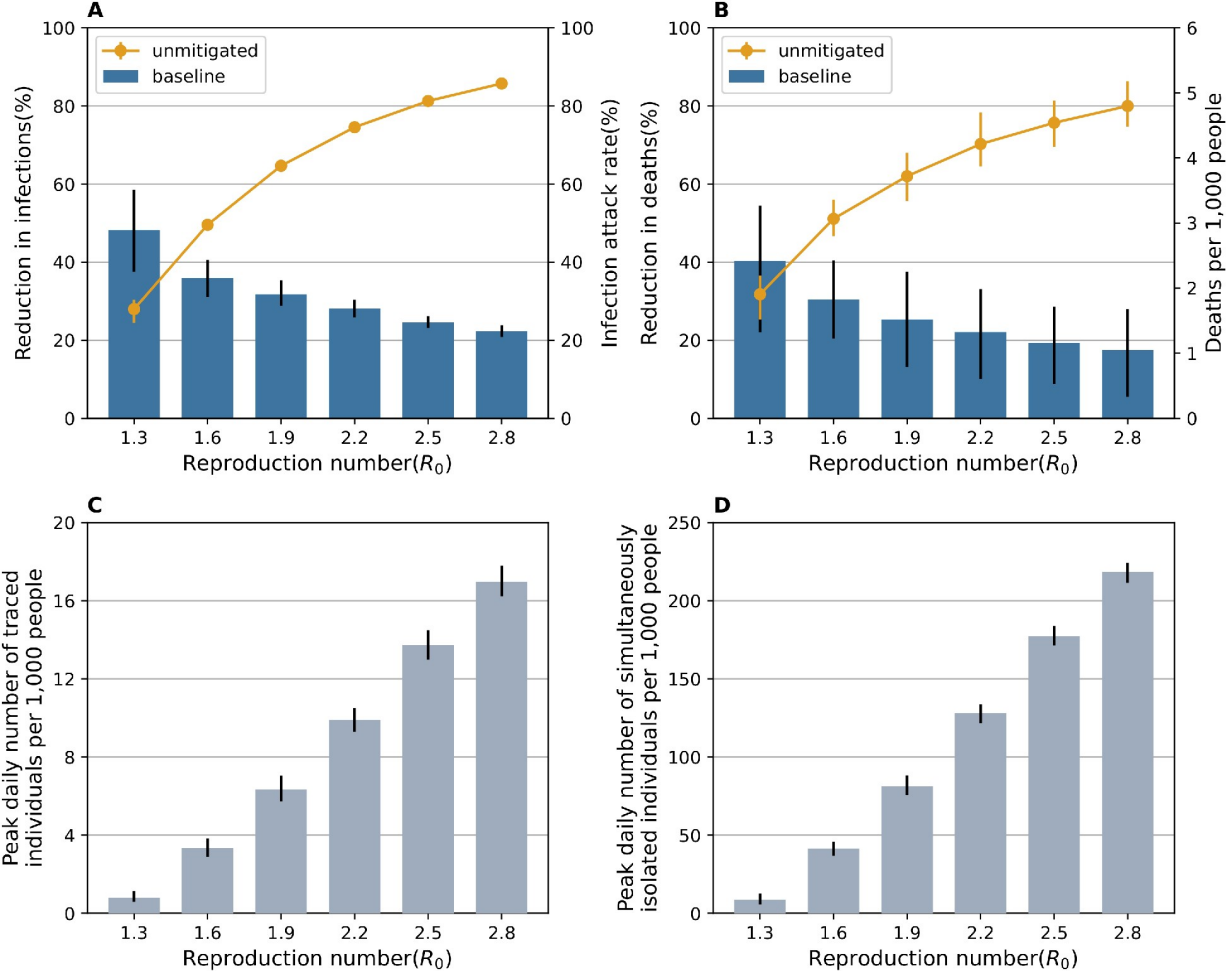
Effectiveness and social/implementation cost of TTI. **A** Infection attack rate (IAR) without TTI strategy (unmitigated) and reduction in the cumulative infections by the Test-Trace-Isolate strategy (baseline) with different reproduction number *R*_0_. The vertical error bars indicate the 95% CI. **B** as in **A**, but for the number of deaths per 1,000 people and reduction in deaths. **C** Peak daily number of traced individuals with positive test results (thus contact tracing is needed) per 1,000 people. **D** Peak daily number of simultaneously isolated individuals per 1,000 people.

Without interventions, the cumulative number of infections (i.e., infection attack rate; IAR) and deaths reached 81.2% (95% CI: 80.8–81.7%) (**Fig 3A**) and 4.5 (95% CI: 4.2–4.9) per 1,000 people (**Fig 3B**), respectively. The proportion of pre-symptomatic transmission was 57.0% (95% CI: 56.3–57.7%), which is similar to the value reported in the analysis of the transmission events in Hunan, China [33]. The Test-Trace-Isolate strategy reduced infections and deaths by 24.5% (95% CI: 23.2–26.4%) and 19.3% (95% CI: 8.7–28.8%), respectively, which is consistent with previous studies [26]. The results for other metrics of disease burden are reported in **S1 Supplementary Methods** (**S10 Fig**). The peak daily number of traced individuals and the peak daily number of simultaneously isolated individuals were 13.7 (95% CI: 13.0–14.5) per 1,000 people (**Fig 3C**), and 177.3 (95% CI: 171.4–184.0) per 1,000 people (**Fig 3D**), respectively.

**Fig 4:**
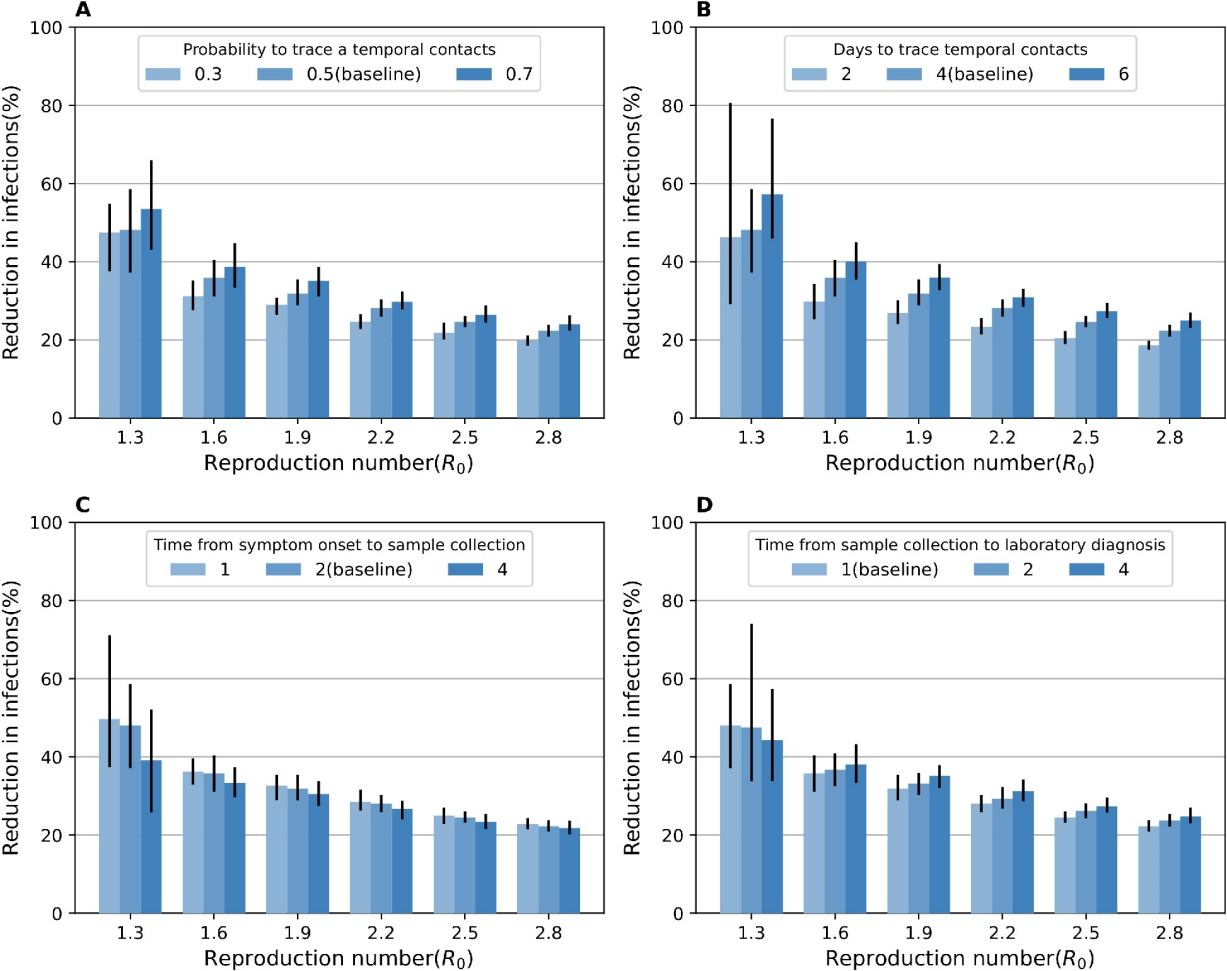
Sensitivity analyses varying the parameters regulating the test and trace processes. **A** Reductions in the cumulative infections with different probabilities that contacts on the temporal layer to be successfully traced. The vertical error bars indicate the 95% CI. **B** as in **A**, but the days to trace contacts on the temporal contact layer before sample collection of the primary cases were varied. **C** as in **A**, but the time from symptom onset to sample collection was varied. **D** as in **A**, but the time from sample collection to laboratory diagnosis was varied.

We run a variety of sensitivity analyses varying the parameters regulating TTI. Two parameters, the probability that contacts on the temporal layer to be successfully traced and the number of days contacts are traced on the temporal contact layer before sample collection of the primary case, are positively associated with a reduction of IAR (**Fig 4A and 4B**), as more infected individuals are isolated. A shorter time interval from symptom onset to sample collection is associated with a higher reduction of IAR (**Fig 4C**), because contacts of tested individuals on the temporal contact layer are promptly disconnected from the network after the sample collection, in agreement in previous studies [7].

**Fig 5:**
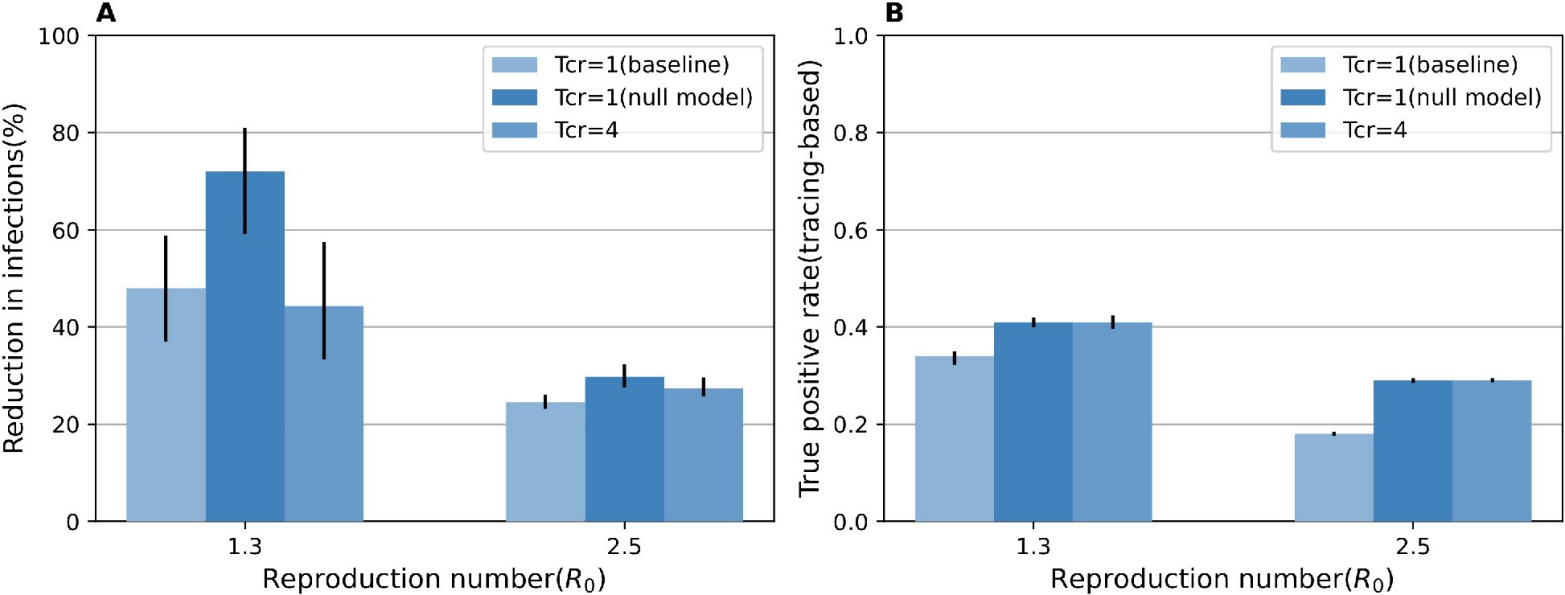
Analysis of baseline with longer delay varying the time from sample collection to laboratory diagnosis. **A** Reduction in the cumulative infections. **B** True positive rate among the traced individuals. “*T*_*cr*_ = 1 (baseline)” and “*T*_*cr*_ = 4” are the models setting the time from sample collection to laboratory diagnosis as 1 day or 4 days, respectively. “*T*_*cr*_ = 1 (null model)” is the model setting the time from sample collection to laboratory diagnosis as 1 day, but the true positive rate among the traced individuals is the same as the model with “*T*_*cr*_ = 4”. Other parameter settings are the same.

The impact of the time interval from sample collection to laboratory diagnosis (*T*_*cr*_) on the reduction of IAR depends on the pathogen transmissibility (*R*_0_) (**Fig 4D**). Longer *T*_*cr*_ decreases the IAR reduction under a low transmissibility scenario (*R*_0_ = 1.3); however, it increases the IAR reduction under a high transmissibility scenario (*R*_0_ = 2.5). This complex association is due to the balance between the probability of secondary transmission from isolated individuals and the probability that secondary infections can be effectively isolated. The former probability decreases with shorter *T*_*cr*_, because their links on the static layer remain active while they wait for the test result. Furthermore, it increases with high transmissibility. Meanwhile, the probability that secondary infections can be effectively isolated decreases with shorter *T*_*cr*_, under which the traced (and infected) individuals may not be properly identified by the test because of low test sensitivity during the early phase of infection (many secondary transmissions occur around symptom onset of primary cases, given that the incubation period [6.3 days] and the generation time [7.0 days] are close). Furthermore, it decreases with high transmissibility because more asymptomatic or pre-symptomatic individuals (lower sensitivity) are tested. Indeed, the observed true positive rate, especially among those identified through contact tracing, was reduced by the high value of *R*_0_ (**S11B and S11D Figs**). To quantify the impact of this higher test positive rate among traced individuals, we designed a null model with the same parameter settings except for the test sensitivity for the pre-symptomatic and asymptomatic individuals, which was set so that the observed true positive rate among traced individuals is the same as for the model with *T*_*cr*_ = 4 (**Fig 5B**). Thus, the difference in IAR reduction between the null and baseline models (27.6% with *R*_0_ = 1.3; **Fig 5A**) is due to the increased true positive rate for traced individuals. This difference between the null model and the model with *T*_*cr*_ = 4 (23.9% with *R*_0_ = 1.3; **Fig 5A**) is associated with a larger number infections occurring while test results are not available yet. Thus, when *R*_0_ is low (i.e., 1.3), IAR is reduced by 3.7%, while it increases by 5.2% when *R*_0_ is high (2.5).

Overall, from our sensitivity analyses, we found that TTI can be implemented more efficiently by increasing the likelihood of tracing contacts or reducing testing delays. However, TTI alone is not sufficient to control an epidemic even in a best-case scenario. To mitigate COVID-19 burden, combining TTI with other interventions would need to be considered. Indeed, during the COVID-19 pandemic, multiple non-pharmaceutical interventions (NPIs), such as mask wearing and improved hygiene practices [4] were implemented along with TTI, the combination of which resulted in a reduction of the transmission risk (and of the effective reproduction number). Here we run simulations for different values of the reproduction number to resemble the effect of different NPIs that are implemented to lower the transmission risk. In addition, we also run simulations with a higher value *R*_0_ (i.e., = 2.8) to account for the variability in *R*_0_ estimates, for instance due to different variants, socio-demographic characteristics, and contact patterns [13]. Our simulations show that TTI is more effective for lower values of *R*_0_. For example, when *R*_0_ is 1.3, TTI can reduce the total number of infections and deaths by 48.1% (95% CI: 37.6–58.5%) and 40.4% (95% CI: 23.5–54.4%), respectively. The social and implementation costs of TTI are reduced for lower *R*_0_ values. For example, when *R*_0_ is 1.3, the peak daily number of individuals to be traced and the peak daily number of simultaneously isolated individuals drop to 0.8 (95% CI: 0.6–1.1) per 1,000 people and 8.8 (95% CI: 5.7–12.5) per 1,000 people, respectively. Therefore, combining TTI with NPIs not only increases the effectiveness of TTI strategy, but also reduces the social and implementation costs associated with TTI.

We further run sensitivity analyses varying the parameters unrelated to TTI, namely: (1) infectiousness of asymptomatic individuals, *χ*(*A*); (2) the number of initially infected individuals; (3) the probability that a symptomatic individual is tested, *P*_*test*_. The number of initially infected individuals had no impact on the effectiveness of TTI (**S13 Fig**); however, lower *χ*(*A*) and higher *P*_*test*_ were associated with higher effectiveness of TTI (**S12A and S14A Figs**) and deaths (**S12B and S14B Figs**).

**Fig 6:**
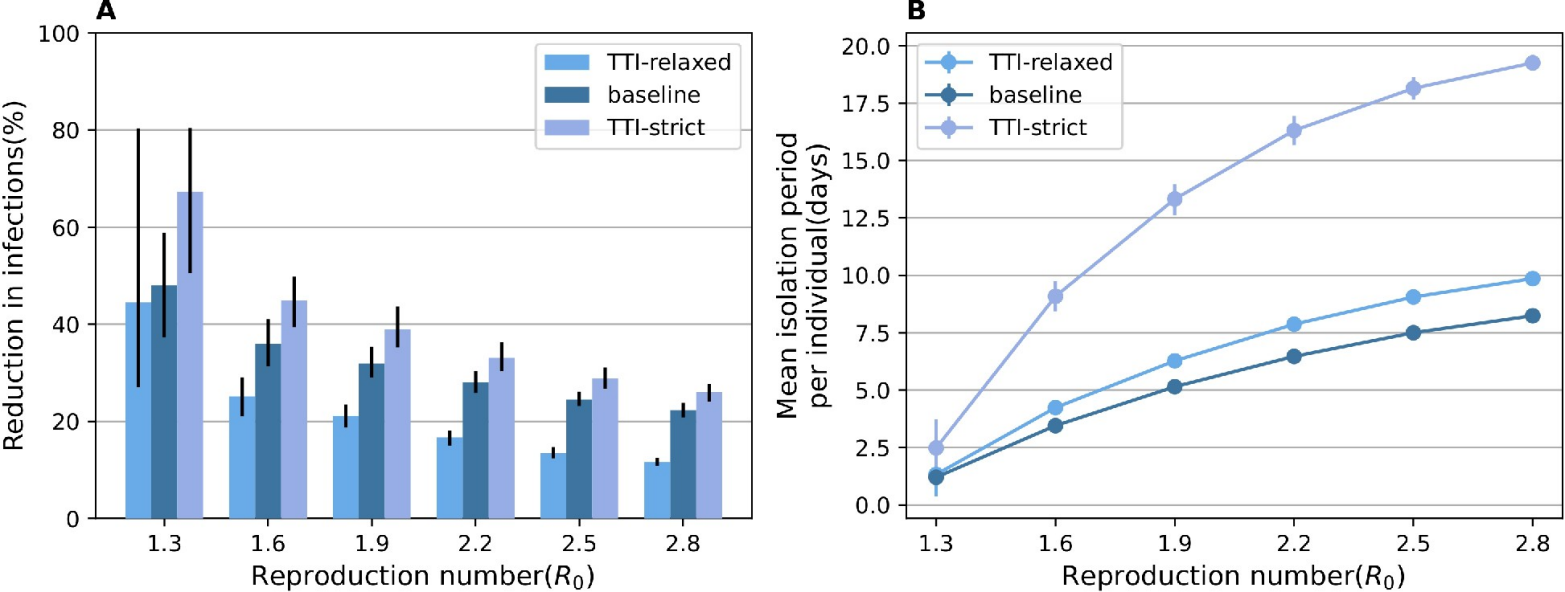
Effectiveness and burden of TTI-relaxed and TTI-strict. **A** Reductions in the cumulative infections with different TTI strategies. (TTI-relaxed) Contacts on both layers remains while the test results are waited. (TTI-strict) Contacts on both layers are disconnected while the test results are waited. **B** as in **A**, but for the mean isolation period.

A point of discussion during the pandemic has been whether individuals should be isolated while waiting for the result of a test [59–61]. In the main analysis, we assumed that only the links on the temporal contact layer are disconnected while waiting for the test result. Here, we run two sensitivity analyses: (1) links on both layers are disconnected (TTI-strict) or (2) all links remain active (TTI-relaxed) while waiting for the test result. The effectiveness of TTI was lower when a less restrictive TTI is considered (**Fig 6A**). Specifically, for *R*_0_ = 2.5, the reduction of the IAR was reduced by 45.3% and increased by 17.6% when considering TTI-relaxed and TTI-strict, respectively (**Fig 6A**), as compared with TTI (baseline). Meanwhile, the mean isolation period was 1.5 days and 10.5 days longer for TTI-relaxed and TTI-strict, respectively (**Fig 6B**). The longer isolation period of TTI-relaxed was due to the larger number of infections, whereas that of TTI-strict is due to its mechanism, as individuals are isolated while waiting for the test result. These findings suggest that unnecessary gatherings and social activities should be avoided while waiting for test results; however, avoiding regular activities (such as going to schools or offices) may not be necessary, considering the trade-offs between the effectiveness of the TTI strategy and its social and implementation costs.

### Reactive distancing policy

We considered to combine TTI with the other interventions by varying *R*_0_ in the sensitivity analysis. However, reduced values of *R*_0_ does not have a practical interpretation as there is no clear connection between the magnitude of reduction in *R*_0_ and specific interventions. Here, we explicitly simulated a reactive social distancing policy as an example. Limiting social events and gathering has been adopted in many countries and regions as a social distancing policy to limit SARS-CoV-2 transmission, especially at early phase of the pandemic.

We analyzed two social distancing policies: 1. reactive social distancing, i.e., limiting unnecessary gathering and events only (a fraction of contacts on temporal contact layer is disconnected) [62], 2. reactive “all-level” distancing, limiting both unnecessary gathering and events and necessary activities (a fraction of contacts on both contact layers is disconnected) [63]. Those policies are triggered when the cumulative number of detected cases exceeds a predefined threshold value. Details are reported in **S1 Supplementary Methods** (6. Reactive distancing policy) and **S3 Table**.

**Fig 7:**
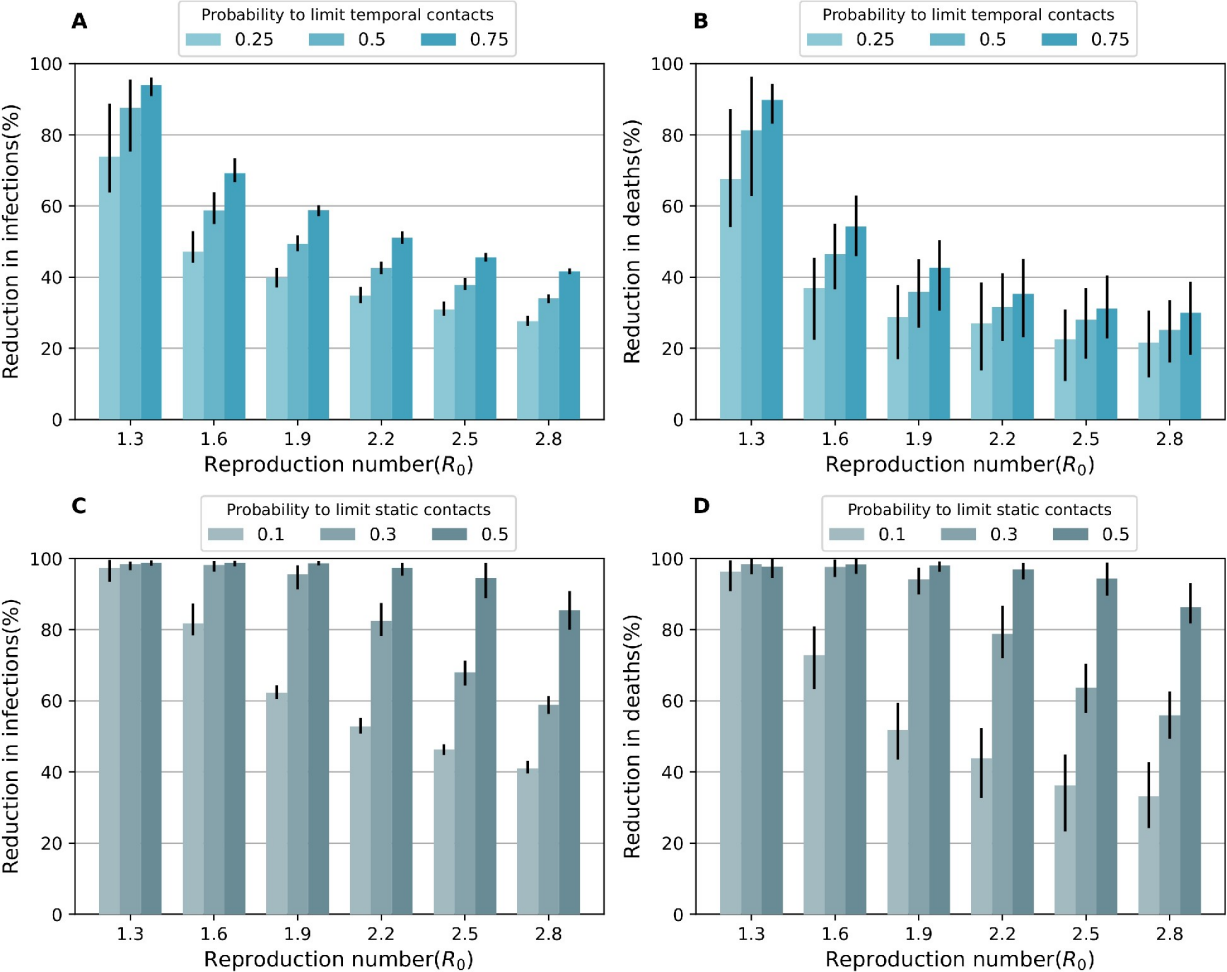
Effectiveness of reactive distancing policy coupled with the TTI strategy. **A** and **B** Reactive social distancing: a fraction of contacts on temporal contact layer are disconnected. **A** for the reduction in the cumulative infections, **B** as in **A**, but for the reduction in deaths. **C** and **D** Reactive all-level distancing: 50% of contacts on temporal layer are disconnected and a fraction of contacts on static contact layer is disconnected. **C** for the reduction in the cumulative infections, **D** as in **C**, but for the reduction in deaths. Those policies are triggered when the cumulative number of detected cases exceeds 50.

We estimate reactive social distancing to have a substantial impact in reducing COVID-19 burden (**Fig 7A and 7B**). For example, when *R*_0_ = 2.5 and 50% or 75% of the contacts on the temporal layers are disconnected (*P*_*social*_ = 0.5 or 0.75), the IAR reduction reached 37.8% (95% CI: 36.4–39.7%) and 45.6% (95% CI: 44.4–46.7%), respectively. Reactive “all-level” distancing further reduced COVID-19 burden (**Fig 7C and 7D**). When 30% of contacts on the static layer and 50% of contacts on the temporal layer are disconnected (*P*_*social*_ = 0.5, *P*_*static*_ = 0.3), 68.1% (95% CI: 64.3–71.3%) of infections could be averted (**Fig 7C**).

## Discussion

Test-Trace-Isolate strategies have been commonly adopted to mitigate and control the spread of infectious diseases [64]. Previous modelling studies have identified several key factors that are crucial to determine the effectiveness of TTI, such as the reproduction number and the proportion of transmissions that occur before symptom onset [13].

On top of those previous studies, we incorporated an essential feature of social behavior shaping the transmission risk. TTI primarily serves to control (in-person) contacts between individuals. However, contacts are highly heterogeneous, and incorporating proper contact patterns in transmission models is essential to assess TTI strategies. Here, we proposed a novel static-temporal multiplex network that incorporates two complementary types of contacts: regular contacts on a static contact layer and random contacts on temporal contact layer. On this comprehensive contact network, we developed a stochastic agent-based SARS-CoV-2 transmission model, which enabled us to implement individual-level interventions accounting for the natural heterogeneity of human contact patterns.

Using our model, we provided a quantitative assessment of the effectiveness of TTI strategies, and found that TTI alone can only partially mitigate COVID-19 burden. This is due to the high proportion of pre-asymptomatic and asymptomatic transmission, which hinders the successful and prompt identification of infected individuals when a symptom-based trigger is considered. When R_0_ is 2.2, given that infectiousness onset almost coincides with symptom onset (infectiousness appears 0.51–0.77 days before symptom onset on average), Peak et al [65]. found that tracing contacts and quarantine or active monitoring individuals triggered by symptom presence have a certain potential to control the SARS-CoV-2 spreading. However, in situations where less resources are available for tracing, or the adherence to such interventions is low, the effectiveness of TTI could be low. For example, if less than 20% of the population follows the rule of self-reporting and isolation due to symptoms or tracing, large outbreaks are unlikely to be avoided even with scaled-up tracing and quick turnaround testing [28]. We further examined reactive social distancing strategies to be combined with TTI. Specifically, we considered two types of reactive social distancing: 1) a moderate one, which limits only non-essential contacts (e.g., gatherings, social events), thus reducing contacts on the temporal contact layer only; 2) and a strict one, which limits not only non-essential contacts but also a part of regular contacts on the static contact layer (similar to what happens when reactive school and workplace closures are adopted). We found that limiting temporal contacts alone could avert less than 50% of the infections, especially when the transmissibility potential is high (*R*_0_≥2.2). Furthermore, limiting both temporal and regular contacts could remarkably enhance the effectiveness of the TTI strategies, as the main transmission route of SARS-CoV-2 transmission is through regular contacts. Thus, especially when effective therapeutics or vaccines were not available, stay-at-home orders to suppress epidemic spread were considered.

Our study has several strengths. First, our model is calibrated using epidemiological and contact survey data. In particular, the structure of the contact network is based on individual-level contact patterns data stratified by age and social setting. However, it is worth noting that, to reconstruct the static-temporal network, we used contact survey data collected before the COVID-19 pandemic. The pandemic has altered the contact patterns of the population in ways that go beyond the implementation of social distancing policies, including individual considerations about the perceived risk of infection [66]. Future studies are needed to estimate contact patterns in the post-pandemic era. Second, we estimated the social and implementation costs of TTI as well as its effectiveness. The social and implementation costs of TTI can be defined in different ways, but further cost-effectiveness analyses are needed to estimate economic costs and broader social implications of TTI strategies as well as other interventions. Finally, our stochastic agent-based model with its static-temporal network structure allows the analysis of individual-level interventions with a high degree of fidelity and flexibility. In this study, we analyzed TTI; however, the model can be used to examine other individual-level NPIs as well as pharmacological interventions, such as vaccination programs, and antiviral treatments and prophylaxis.

Our study has several limitations. First, we grouped contacts that take place in the household, school, and workplace settings into a single layer (i.e., static contact). Therefore, our network does not explicitly replicate the structure of each household, school, and workplace. As such, our network cannot capture the saturation effect of household transmission observed in previous studies [67,68], which may lead our model to slightly overestimate the infection attack rate. Moreover, we did not consider heterogeneity in the transmission risk for contacts on the same layer. For example, the transmission risk of contacts between household members may differ from that of contacts between classmates, and transmission may also differ between individuals due to different behaviors. Further studies are needed to investigate the granularity of contact patterns that is needed to capture epidemiological dynamics with/without interventions. Second, in our simulations, we assumed complete connection among people attending the same social event, which might not reflect the reality. Contacts may also depend on types of individual (not just age, as considered in this study). The assumption of full connectivity may increase the risk of pathogen transmission among individuals in the same group, as each susceptible individual in that group can be infected by any infectious member of the group. Third, our simulations assumed an instantaneous turnaround time for contact tracing, but contact tracing is often implemented through interviews conducted by an operator; thus, it may take several days to gather all information and identify contacts. Kretzschmar et al. ‘s study [69] have shown that the tracing delay has certain impact on the contact tracing effectiveness, although lower than the testing delay. Thus, the assumption of an instantaneous turnaround time for contact tracing may lead to a slightly overestimation of the effectiveness of TTI. Fourth, we did not consider the possibility of false positive test results. Although rare, false positive results can increase the social and implementation costs of TTI. This aspect can be particularly relevant for cost-effectiveness analyses.

### Conclusion

In conclusion, we proposed a novel static-temporal multiplex network that captures contact patterns. Using this model, we examined the effectiveness and the social/implementation costs of TTI and identify key parameters regulating its effectiveness. We found that TTI alone could not control a COVID-19 outbreak. The infection attack rate can however be reduced by 24.5% when R_0_ = 2.5; when NPIs are implemented to lower the transmission risk (e.g., R_0_ = 1.3), about 48.3% of the infections can be averted. Our findings highlight the importance of combining TTI with an overall reduction of transmission obtained by limiting contacts regardless of their types, given the substantial proportion of asymptomatic and pre-symptomatic transmission and high reproduction number of the ancestral SARS-CoV-2 lineages as well as subsequent variants.

## Supporting information

S1 Supplementary Methods 1. Contact survey data for the static-temporal multiplex network. 2. Static-Temporal Multiplex Network. 3. SARS-CoV-2 Transmission model. 4. Calibration. 5. The Test-Trace-Isolate strategy. 6. Reactive distancing policy.
(DOCX)

S1 Fig The age-group-dependent contact frequency of individual contacts from 0 to 39. (TIF)

S2 Fig The age-group-dependent contact frequency of individual contacts over 40. (TIF)

S3 Fig The Gaussian kernel fitted age-group-dependent age distribution of contactees of individual contacts from 0 to 39. (TIF)

S4 Fig The Gaussian kernel fitted age-group-dependent age distribution of contactees of individual contacts over 40. (TIF)

S5 Fig The Gaussian kernel fitted age-group-dependent age distribution of participants in gathering and events from 0 to 39. (TIF)

S6 Fig The Gaussian kernel fitted age-group-dependent age distribution of participants in gathering and events over 40. (TIF)

S7 Fig Contact matrix representing the static contact layer. Colors represent the mean number of contacts between an individual in given age group with individuals with any age group.
(TIF)

S8 Fig Contact matrix representing the temporal contact layer. The panels show two realizations of the temporal contact layers for two randomly selected day of a simulation. Colors represent the mean number of contacts between an individual in given age group with individuals with any age group.
(TIF)

S9 Fig SARS-CoV-2 transmission model. The detailed description of symbols in the figure referred to **S1 Table.**
(TIF)

S10 Fig Disease burdens under the baseline TTI strategy. **A.** The number of symptomatic infections per 1,000 people without the TTI strategy (orange dots) and the reduction by the baseline TTI strategy (blue bars). Reproduction numbers (*R*_0_) are varied. The vertical error bars indicate the 95% CI. **B.** The same as **A**, but using the number of hospitalization as the disease burden. **C.** The same as **A**, but using the number of ICU patients as the disease burden.
(TIF)

S11 Fig Impact of the time from sample collection to laboratory diagnosis. **A.** Average number of secondary infection (per a primary case) while waiting for the test results. **B.** observed true positive rate. **C.** as in **B**, but only for the symptomatic individuals. **D.** as in **B**, but only for those recruited through contact tracing.
(TIF)

S12 Fig Impact of the relative infectiousness of asymptomatic individuals. **A.** Reduction in the cumulative infections under different reproduction number and the relative infectiousness of asymptomatic cases. The vertical error bars indicate the 95% CI. **B.** as in **A**, but reduction in deaths.
(TIF)

S13 Fig Impact of the number of initial infected individuals. A. Reduction in the cumulative infections under different reproduction number and the initial number of cases. The vertical error bars indicate the 95% CI. **B.** as in **A**, but reduction in deaths.
(TIF)

S14 Fig Impact of the probability to test a symptomatic individual. **A.** Reduction in the cumulative infections under different reproduction number and the probability to test a symptomatic individual. The vertical error bars indicate the 95% CI. **B.** as in **A**, but reduction in deaths.
(TIF)

S15 Fig Impact of the reactive threshold values. **A.** Reduction in the cumulative infections under different reproduction number and the reactive threshold values for the reactive social distancing. The vertical error bars indicate the 95% CI. **B.** as in **A**, but reduction in deaths. **C.** as in **A**, but for the reactive all-level distancing. **D.** as in **C**, but reduction in deaths.
(TIF)

S16 Fig Daily new infections and new deaths. A. and **B.** for the daily new infections and daily new deaths under the unmitigated scenario (without TTI) with reproduction number of R_0_ = 1.3, 2.5 and 2.8, the shadows indicate the 95% CI; **C**. and **D.** for baseline where TTI is implemented under the scenario that the reactive social distancing is triggered when the cumulative number of detected cases exceeds 50, specifically, 50% of contacts on the temporal layer are disconnected, and under the scenario that the reactive all-level distancing is triggered when the cumulative number of detected cases exceeds 50, specifically, 50% of contacts on the temporal layer are disconnected and 30% of contacts on the static contact layer are disconnected. R_0_ = 2.5. **E.** and **F.** as in **C.** and **D.**, but R_0_ = 2.8.
(TIF)

S1 Table Parameters for SARS-Cov-2 transmission model and disease burden. (DOCX)

S2 Table Parameters for TTI. (DOCX)

S3 Table Parameters for reactive distancing policies. (DOCX)

## References

[1] WHO COVID-19 Dashboard. Geneva: World Health Organization, 2020. Available online: https://covid19.who.int/ (2022)

[2] Kaye AD, Okeagu CN, Pham AD, Silva RA, Hurley JJ, Arron BL, et al. Economic impact of COVID-19 pandemic on healthcare facilities and systems: International perspectives. Best Practice & Research. 2021;35(3):293–306. doi: 10.1016/j.bpa.2020.11.009 34511220 PMC7670225

[3] Lau H, Khosrawipour V, Kocbach P, Mikolajczyk A, Schubert J, Bania J, et al. The positive impact of lockdown in Wuhan on containing the COVID-19 outbreak in China. Journal of Travel Medicine. 2020;27(3). doi: 10.1093/jtm/taaa037 32181488 PMC7184469

[4] Flaxman S, Mishra S, Gandy A, Unwin HJT, Mellan TA, Coupland H, et al. Estimating the effects of non-pharmaceutical interventions on COVID-19 in Europe. Nature. 2020;584(7820):257–61. doi: 10.1038/s41586-020-2405-7 32512579

[5] Olivier C, Yuriy G, Michael W. The Cost of the Covid-19 Crisis: Lockdowns, Macroeconomic Expectations, and Consumer Spending. National Bureau of Economic Research. 2020. 10.3386/w27141

[6] Di Domenico L, Pullano G, Sabbatini CE, Boelle PY, Colizza V. Impact of lockdown on COVID-19 epidemic in Ile-de-France and possible exit strategies. BMC Medicine. 2020;18(1):240. doi: 10.1186/s12916-020-01698-4 32727547 PMC7391016

[7] Liu QH, Zhang J, Peng C, Litvinova M, Huang S, Poletti P, et al. Model-based evaluation of alternative reactive class closure strategies against COVID-19. Nature Communications. 2022;13(1):322. doi: 10.1038/s41467-021-27939-5 35031600 PMC8760266

[8] United Nations Educational. School closure information because of COVID-19. https://en.unesco.org/covid19/educationresponse (2020).

[9] Di Domenico L, Pullano G, Sabbatini CE, Boelle PY, Colizza V. Modelling safe protocols for reopening schools during the COVID-19 pandemic in France. Nature Communications. 2021;12(1):1073. doi: 10.1038/s41467-021-21249-6 33594076 PMC7887250

[10] Munday JD, Sherratt K, Meakin S, Endo A, Pearson CAB, Hellewell J, et al. Implications of the school-household network structure on SARS-CoV-2 transmission under school reopening strategies in England. Nature Communications. 2021;12(1):1942. doi: 10.1038/s41467-021-22213-0 33782396 PMC8007691

[11] Black JRM, Bailey C, Przewrocka J, Dijkstra KK, Swanton C. COVID-19: the case for health-care worker screening to prevent hospital transmission. Lancet. 2020;395(10234):1418–20. doi: 10.1016/S0140-6736(20)30917-X 32305073 PMC7162624

[12] Smith DRM, Duval A, Zahar JR, Opatowski L, Temime L, Nosocomial E-MWG. Rapid antigen testing as a reactive response to surges in nosocomial SARS-CoV-2 outbreak risk. Nature Communications. 2022;13(1). doi: 10.1038/s41467-021-27845-w 35017499 PMC8752617

[13] Hellewell J, Abbott S, Gimma A, Bosse NI, Jarvis CI, Russell TW, et al. Feasibility of controlling COVID-19 outbreaks by isolation of cases and contacts. Lancet Global Health. 2020;8(4):e488–e96. doi: 10.1016/S2214-109X(20)30074-7 32119825 PMC7097845

[14] Kucharski AJ, Klepac P, Conlan AJK, Kissler SM, Tang ML, Fry H, et al. Effectiveness of isolation, testing, contact tracing, and physical distancing on reducing transmission of SARS-CoV-2 in different settings: a mathematical modelling study. Lancet Infectious Diseases. 2020;20(10):1151–60. doi: 10.1016/S1473-3099(20)30457-6 32559451 PMC7511527

[15] Gosce L, Phillips PA, Spinola P, Gupta DRK, Abubakar PI. Modelling SARS-COV2 Spread in London: Approaches to Lift the Lockdown. Journal of Infection. 2020;81(2):260–5. doi: 10.1016/j.jinf.2020.05.037 32461062 PMC7246004

[16] Aleta A, Martin-Corral D, Pastore YPA, Ajelli M, Litvinova M, Chinazzi M, et al. Modelling the impact of testing, contact tracing and household quarantine on second waves of COVID-19. Nature Human Behaviour. 2020;4(9):964–71. doi: 10.1038/s41562-020-0931-9 32759985 PMC7641501

[17] Glasser JW, Hupert N, McCauley MM, Hatchett R. Modeling and public health emergency responses: lessons from SARS. Epidemics. 2011;3(1):32–7. doi: 10.1016/j.epidem.2011.01.001 21420657 PMC7105002

[18] Eames KT, Webb C, Thomas K, Smith J, Salmon R, Temple JM. Assessing the role of contact tracing in a suspected H7N2 influenza A outbreak in humans in Wales. BMC Infectious Diseases. 2010;10:141. doi: 10.1186/1471-2334-10-141 20509927 PMC2890003

[19] Swanson KC, Altare C, Wesseh CS, Nyenswah T, Ahmed T, Eyal N, et al. Contact tracing performance during the Ebola epidemic in Liberia, 2014–2015. PLoS Neglected Tropical Diseases. 2018;12(9):e0006762. doi: 10.1371/journal.pntd.0006762 30208032 PMC6152989

[20] Keeling MJ, Hollingsworth TD, Read JM. Efficacy of contact tracing for the containment of the 2019 novel coronavirus (COVID-19). Journal of Epidemiology and Community Health. 2020;74(10):861–6. doi: 10.1136/jech-2020-214051 32576605 PMC7307459

[21] Bi QF, Wu YS, Mei SJ, Ye CF, Zou X, Zhang Z, et al. Epidemiology and transmission of COVID-19 in 391 cases and 1286 of their close contacts in Shenzhen, China: a retrospective cohort study. Lancet Infectious Diseases. 2020;20(8):911–9. doi: 10.1016/S1473-3099(20)30287-5 32353347 PMC7185944

[22] Chen YH, Fang CT, Huang YL. Effect of Non-lockdown Social Distancing and Testing-Contact Tracing During a COVID-19 Outbreak in Daegu, South Korea, February to April 2020: A Modeling Study. International Journal of Infectious Diseases. 2021;110:213–21. doi: 10.1016/j.ijid.2021.07.058 34333119 PMC8320402

[23] Baker MG, Kvalsvig A, Verrall AJ, Telfar-Barnard L, Wilson N. New Zealand’s elimination strategy for the COVID-19 pandemic and what is required to make it work. New Zealand Medical Journal. 2020;133(1512):10–4. 32242173

[24] Wu JH, Tang B, Bragazzi NL, Nah K, McCarthy Z. Quantifying the role of social distancing, personal protection and case detection in mitigating COVID-19 outbreak in Ontario, Canada. Journal of Mathematics in Industry. 2020;10(1). doi: 10.1186/s13362-020-00083-3 32501416 PMC7249976

[25] Pullano G, Di Domenico L, Sabbatini CE, Valdano E, Turbelin C, Debin M, et al. Underdetection of cases of COVID-19 in France threatens epidemic control. Nature. 2020; doi: 10.1038/s41586-020-03095-6 33348340

[26] Contreras S, Dehning J, Loidolt M, Zierenberg J, Spitzner FP, Urrea-Quintero JH, et al. The challenges of containing SARS-CoV-2 via test-trace-and-isolate. Nature Communications. 2021;12(1):378. doi: 10.1038/s41467-020-20699-8 33452267 PMC7810722

[27] Chiu WA, Fischer R, Ndeffo-Mbah ML. State-level needs for social distancing and contact tracing to contain COVID-19 in the United States. Nature Human Behaviour. 2020;4(10):1080–+. doi: 10.1038/s41562-020-00969-7 33024280 PMC7572893

[28] Davis EL, Lucas TCD, Borlase A, Pollington TM, Abbott S, Ayabina D, et al. Contact tracing is an imperfect tool for controlling COVID-19 transmission and relies on population adherence. Nature Communications. 2021;12(1). doi: 10.1038/s41467-021-25531-5 34518525 PMC8438018

[29] Fraser C, Riley S, Anderson RM, Ferguson NM. Factors that make an infectious disease outbreak controllable. Proceedings of the National Academy of Sciences of the United States of America. 2004;101(16):6146–51. doi: 10.1073/pnas.0307506101 15071187 PMC395937

[30] Oran DP, Topol EJ. Prevalence of Asymptomatic SARS-CoV-2 Infection A Narrative Review. Annals of Internal Medicine. 2020;173(5):362–+. doi: 10.7326/M20-3012 32491919 PMC7281624

[31] Moghadas SM, Fitzpatrick MC, Sah P, Pandey A, Shoukat A, Singer BH, et al. The implications of silent transmission for the control of COVID-19 outbreaks. Proceedings of the National Academy of Sciences of the United States of America. 2020;117(30):17513–17515. doi: 10.1073/pnas.2008373117 32632012 PMC7395516

[32] Sun K, Wang W, Gao L, Wang Y, Luo K, Ren L, et al. Transmission heterogeneities, kinetics, and controllability of SARS-CoV-2. Science. 2021;371(6526). doi: 10.1126/science.abe2424 33234698 PMC7857413

[33] Hu S, Wang W, Wang Y, Litvinova M, Luo K, Ren L, et al. Infectivity, susceptibility, and risk factors associated with SARS-CoV-2 transmission under intensive contact tracing in Hunan, China. Nature Communications. 2021;12(1):1533. doi: 10.1038/s41467-021-21710-6 33750783 PMC7943579

[34] He X, Lau EHY, Wu P, Deng X, Wang J, Hao X, et al. Temporal dynamics in viral shedding and transmissibility of COVID-19. Nature Medicine. 2020;26(5):672–5. doi: 10.1038/s41591-020-0869-5 32296168

[35] Bauch CT, Galvani AP. Epidemiology. Social factors in epidemiology. Science. 2013;342(6154):47–9. doi: 10.1126/science.1244492 24092718 PMC4229037

[36] Leung NHL. Transmissibility and transmission of respiratory viruses. Nature Reviews Microbiology. 2021;19(8):528–45. doi: 10.1038/s41579-021-00535-6 33753932 PMC7982882

[37] Pitol AK, Julian TR. Community Transmission of SARS-CoV-2 by Surfaces: Risks and Risk Reduction Strategies. Environmental Science & Technology Letters. 2021;8(3):263–9. doi: 10.1021/acs.estlett.0c00966 37566313

[38] Harvey AP, Fuhrmeister ER, Cantrell ME, Pitol AK, Swarthout JM, Powers JE, et al. Longitudinal Monitoring of SARS-CoV-2 RNA on High-Touch Surfaces in a Community Setting. Environmental Science & Technology Letters. 2021;8(2):168–75. doi: 10.1021/acs.estlett.0c00875 34192125 PMC7927285

[39] Garry M, Hope L, Zajac R, Verrall AJ, Robertson JM. Contact Tracing: A Memory Task With Consequences for Public Health. Perspectives on Psychological Science. 2021;16(1):175–87. doi: 10.1177/1745691620978205 33301692

[40] Louail T, Lenormand M, Picornell M, Garcia Cantu O, Herranz R, Frias-Martinez E, et al. Uncovering the spatial structure of mobility networks. Nature Communications. 2015;6:6007. doi: 10.1038/ncomms7007 25607690

[41] Sun L, Axhausen KW, Lee DH, Huang X. Understanding metropolitan patterns of daily encounters. Proceedings of the National Academy of Sciences of The United States Of America. 2013;110(34):13774–9. doi: 10.1073/pnas.1306440110 23918373 PMC3752247

[42] Zhang MX, Verbraeck A, Meng RQ, Chen B, Qiu XG. Modeling Spatial Contacts for Epidemic Prediction in a Large-scale Artificial City. Journal of Artificial Societies and Social Simulation. 2016;19(4). 10.18564/jasss.3148

[43] Mistry D, Litvinova M, Pastore YPA, Chinazzi M, Fumanelli L, Gomes MFC, et al. Inferring high-resolution human mixing patterns for disease modeling. Nature Communications. 2021;12(1):323. doi: 10.1038/s41467-020-20544-y 33436609 PMC7803761

[44] Litvinova M, Liu QH, Kulikov ES, Ajelli M. Reactive school closure weakens the network of social interactions and reduces the spread of influenza. Proceedings of the National Academy of Sciences of The United States Of America. 2019;116(27):13174–81. doi: 10.1073/pnas.1821298116 31209042 PMC6613079

[45] Mousa A, Winskill P, Watson OJ, Ratmann O, Monod M, Ajelli M, et al. Social contact patterns and implications for infectious disease transmission—a systematic review and meta-analysis of contact surveys. Elife. 2021;10. doi: 10.7554/eLife.70294 34821551 PMC8765757

[46] Zhang J, Klepac P, Read JM, Rosello A, Wang X, Lai S, et al. Patterns of human social contact and contact with animals in Shanghai, China. Scientific Reports. 2019;9(1):15141. doi: 10.1038/s41598-019-51609-8 31641189 PMC6805924

[47] Liu J, Liao X, Qian S, Yuan J, Wang F, Liu Y, et al. Community Transmission of Severe Acute Respiratory Syndrome Coronavirus 2, Shenzhen, China, 2020. Emerging Infectious Diseases. 2020;26(6):1320–3. doi: 10.3201/eid2606.200239 32125269 PMC7258448

[48] Aleta A, Martin-Corral D, Bakker MA, Pastore YPA, Ajelli M, Litvinova M, et al. Quantifying the importance and location of SARS-CoV-2 transmission events in large metropolitan areas. Proceedings of the National Academy of Sciences of The United States Of America. 2022;119(26):e2112182119. doi: 10.1073/pnas.2112182119 35696558 PMC9245708

[49] Grantz KH, Lee EC, D’Agostino McGowan L, Lee KH, Metcalf CJE, Gurley ES, et al. Maximizing and evaluating the impact of test-trace-isolate programs: A modeling study. PLoS Medicine. 2021;18(4):e1003585. doi: 10.1371/journal.pmed.1003585 33930019 PMC8121292

[50] Moon SA, Scoglio CM. Contact tracing evaluation for COVID-19 transmission in the different movement levels of a rural college town in the USA. Scientific Reports. 2021;11(1):4891. doi: 10.1038/s41598-021-83722-y 33649364 PMC7921112

[51] Bansal S, Read J, Pourbohloul B & Meyers LA. The dynamic nature of contact networks in infectious disease epidemiology. Journal of Biological Dynamics. 2010; 4:5, 478–489, doi: 10.1080/17513758.2010.503376 22877143

[52] Firth JA, Hellewell J, Klepac P, Kissler S, CMMID COVID-19 Working Group; Kucharski AJ, Spurgin LG. Using a real-world network to model localized COVID-19 control strategies. Nature Medicine. 2020;26(10):1616–1622. doi: 10.1038/s41591-020-1036-8 32770169

[53] Vernon MC, Keeling MJ. Representing the UK’s cattle herd as static and dynamic networks. Proceedings. Biological Sciences. 2009;276(1656):469–76. doi: 10.1098/rspb.2008.1009 18854300 PMC2592553

[54] Perez L, Dragicevic S. An agent-based approach for modeling dynamics of contagious disease spread. International Journal of Health Geographics. 2009;8:50. doi: 10.1186/1476-072X-8-50 19656403 PMC2729742

[55] Mossong J, Hens N, Jit M, Beutels P, Auranen K, Mikolajczyk R, Massari M, Salmaso S, Tomba GS, Wallinga J, Heijne J, Sadkowska-Todys M, Rosinska M, Edmunds WJ. Social contacts and mixing patterns relevant to the spread of infectious diseases. PLoS Medicine. 2008; 5(3):e74. doi: 10.1371/journal.pmed.0050074 18366252 PMC2270306

[56] Yang J, Marziano V, Deng X, Guzzetta G, Zhang J, Trentini F, et al. Despite vaccination, China needs non-pharmaceutical interventions to prevent widespread outbreaks of COVID-19 in 2021. Nature Human Behavior. 2021;5(8):1009–20. doi: 10.1038/s41562-021-01155-z 34158650 PMC8373613

[57] Aleta A, Ferraz de Arruda G, Moreno Y. Data-driven contact structures: From homogeneous mixing to multilayer networks. PLoS Computational Biology. 2020;16(7):e1008035. doi: 10.1371/journal.pcbi.1008035 32673307 PMC7386617

[58] Xiao AT, Tong YX, Gao C, Zhu L, Zhang YJ, Zhang S. Dynamic profile of RT-PCR findings from 301 COVID-19 patients in Wuhan, China: A descriptive study. Journal of Clinical Virology. 2020;127:104346. doi: 10.1016/j.jcv.2020.104346 32361324 PMC7151472

[59] Wilder-Smith A, Freedman DO. Isolation, quarantine, social distancing and community containment: pivotal role for old-style public health measures in the novel coronavirus (2019-nCoV) outbreak. Journal of Travel Medicine. 2020;27(2). doi: 10.1093/jtm/taaa020 32052841 PMC7107565

[60] Chen PJ, Mao LJ, Nassis GP, Harmer P, Ainsworth BE, Li FZ. Coronavirus disease (COVID-19): The need to maintain regular physical activity while taking precautions. Journal of Sport and Health Science. 2020;9(2):103–4. doi: 10.1016/j.jshs.2020.02.001 32099716 PMC7031771

[61] Samieefar N, Yari Boroujeni R, Jamee M, Lotfi M, Golabchi MR, Afshar A, et al. Country Quarantine During COVID-19: Critical or Not? Disaster Medicine and Public Health Preparedness. 2021;15(4):e24–e5. doi: 10.1017/dmp.2020.384 33046176 PMC7737116

[62] Brauner JM, Mindermann S, Sharma M, Johnston D, Salvatier J, Gavenciak T, et al. Inferring the effectiveness of government interventions against COVID-19. Science. 2021;371(6531). doi: 10.1126/science.abd9338 33323424 PMC7877495

[63] Bo Y, Guo C, Lin C, Zeng Y, Li HB, Zhang Y, et al. Effectiveness of non-pharmaceutical interventions on COVID-19 transmission in 190 countries from 23 January to 13 April 2020. International Journal of Infectious Diseases. 2021;102:247–53. doi: 10.1016/j.ijid.2020.10.066 33129965 PMC7598763

[64] Eames KT, Keeling MJ. Contact tracing and disease control. Proceedings of the Royal Society B: Biological Sciences. 2003;270(1533):2565–71. doi: 10.1098/rspb.2003.2554 14728778 PMC1691540

[65] Peak CM, Kahn R, Grad YH, Childs LM, Li R, Lipsitch M, et al. Individual quarantine versus active monitoring of contacts for the mitigation of COVID-19: a modelling study. Lancet Infectious Diseases. 2020;20(9):1025–1033. doi: 10.1016/S1473-3099(20)30361-3 32445710 PMC7239635

[66] Zhang J, Litvinova M, Liang Y, Wang Y, Wang W, Zhao S, et al. Changes in contact patterns shape the dynamics of the COVID-19 outbreak in China. Science. 2020;26;368(6498):1481–1486. doi: 10.1126/science.abb8001 32350060 PMC7199529

[67] Liu QH, Ajelli M, Aleta A, Merler S, Moreno Y, Vespignani A. Measurability of the epidemic reproduction number in data-driven contact networks. Proceedings of the National Academy of Sciences of the United States of America. 2018;115(50):12680–12685. doi: 10.1073/pnas.1811115115 30463945 PMC6294899

[68] Scalia Tomba G, Svensson A, Asikainen T, Giesecke J. Some model based considerations on observing generation times for communicable diseases. Mathematical Biosciences. 2010;223(1):24–31. doi: 10.1016/j.mbs.2009.10.004 19854206

[69] Kretzschmar ME, Rozhnova G, Bootsma MCJ, van Boven M, van de Wijgert JHHM, Bonten MJM. Impact of delays on effectiveness of contact tracing strategies for COVID-19: a modelling study. Lancet Public Health. 2020;5(8):e452–e459. doi: 10.1016/S2468-2667(20)30157-2 32682487 PMC7365652

