# Supplementary material for: Evaluating the impact of test-trace-isolate for COVID-19 management and alternative strategies
Source: PLoS Comput Biol. 2023 Sep 1;19(9):e1011423. doi: 10.1371/journal.pcbi.1011423 (PMC10501547; doi:10.1371/journal.pcbi.1011423)
Supplement: S1 Supplementary Methods — 2. Static-Temporal Multiplex Network. 3. SARS-CoV-2 Transmission model. 4. Calibration. 5. The Test-Trace-Isolate strategy. 6. Reactive distancing policy. (DOCX) [file pcbi.1011423.s001.docx]

**Evaluating the impact of Test-Trace-Isolate for COVID-19 management and alternative strategies**

Kun Zhang^1^, Zhichu Xia^2^, Shudong Huang^1^, Gui-Quan Sun^3,4^, Jiancheng Lv^1^, Marco Ajelli^5,#^ ,Keisuke Ejima^6,#,*^, Quan-Hui Liu^1,#,*^

1 College of Computer Science, Sichuan University, Chengdu, China

2 Glasgow College, University of Electronic Science and Technology of China, Chengdu, China

3 Department of Mathematics, North University of China, Taiyuan, China

4 Complex Systems Research Center, Shanxi University, Taiyuan, China

5 Laboratory for Computational Epidemiology and Public Health, Department of Epidemiology and Biostatistics, School of Public Health, Indiana University Bloomington, Bloomington, Indiana, United States of America

6 Lee Kong Chian School of Medicine, Nanyang Technological University, Singapore, Singapore

*Corresponding authors

^#^These authors are joint senior authors on this work.

**Supplementary methods**

[1. Contact survey data for the static-temporal multiplex network 2](#_Toc22371)

[2. Static-Temporal Multiplex Network 3](#_Toc30182)

[3. SARS-CoV-2 Transmission model 4](#_Toc10947)

[4. Calibration 5](#_Toc97)

[5. The Test-Trace-Isolate strategy 6](#_Toc17447)

[6. Reactive distancing policy 7](#_Toc6808)

[7. Additional results 8](#_Toc15531)

[7.1 Additional disease burden results for the baseline analysis 8](#_Toc1580)

[7.2 Sensitivity analysis varying the time from sample collection to laboratory diagnosis. 8](#_Toc31403)

[7.3 Sensitivity analysis varying infectiousness of asymptomatic individuals relative to symptomatic individuals 8](#_Toc18843)

[7.4 Sensitivity analysis varying the number of initially infected individuals 8](#_Toc14645)

[7.5 Sensitivity analysis varying the probability to test a symptomatic individual 8](#_Toc28695)

[7.6 Sensitivity analysis varying the number of detected cases that trigger the reactive distancing policy 9](#_Toc10636)

[7.7 Daily new infections and new deaths 9](#_Toc21840)

[Reference 10](#_Toc12877)

List of Figures.......................................................................................................................................................................11

List of Tables........................................................................................................................................................................12

## Contact survey data for the static-temporal multiplex network

The static-temporal multiplex network model, composed of the static contact layer and the temporal contact layer, was developed to preserve the characteristics of contact patterns observed in a contact surveillance carried out between December 2017 and May 2018 in Shanghai, China [1].

On the static contact layer, contacts are static during the simulation of an epidemic, reflecting the contacts occur among the household members, schoolmates, and colleagues, which correspond to the individual contacts reported in the surveillance. The individual contacts are recorded as skin-to-skin contacts or face-to-face conversation with three or more words exchanged [1]. More than 90% of the individual contacts recorded are occurred in households, workplaces, and schools. The links on the static contact layer were generated using the two distributions informed by the individual contacts reported in the survey:

- $P_{static}^{\alpha}(k)$: age-group-dependent contact frequency (the probability of an individual in age group $\alpha$ has $k$ individual contacts) (**S1 Fig** and **S2 Fig**)
- $P_{static}^{\alpha}$($\beta$): age-group-dependent age distribution of contactees (the probability of an individual in age group $\alpha$ has an individual contact with an individual of age group $\beta$) (**S3 Fig** and **S4 Fig**)

On the temporal contact layer, contacts are updated frequently (everyday) over the epidemic period, reflecting the contacts occur in gathering or social events, which correspond to participated events reported in the surveillance. Contacts in gathering or social events are recorded as contacts with a group with more than 20 individuals [1]. Such group contacts could occur among those who participate gathering or events where a lot of people meet at the same time. Notably, group contacts occur temporarily and randomly, and each contact is hard to directly record. The links on the temporal contact layer were generated using the three distributions informed by the groups contacts reported in the survey:

- $P_{temp}^{\alpha}$: age-group-dependent probability of joining gathering or events (the proportion of individuals in age group $\alpha$ joining gathering and events) (**Fig 1C**)
- $P_{temp}(k)$: event size distribution (the proportion of gathering and events $k$ individuals join) (**Fig 1D**)
- $P_{temp}^{\alpha}(\beta)$: age-group-dependent age distribution of participants in gathering and events (the proportion of attendee’s age group $\beta$ in gathering and events that an individual in age group $\alpha$ joins) (**S5 Fig** and **S6 Fig**).

Age groups were separated by 5-year interval: $\alpha\in\left\{ \left[ 0,4 \right], \left[ 5,9 \right], \left[ 10,14 \right], \ldots, \left[ 70,74 \right],[75,+) \right\}$. All the distributions defined above are smoothed by Gaussian kernels before used for network development.

## Static-Temporal Multiplex Network

We developed a novel static-temporal multiplex network to describe the regular contact pattern and random contact pattern of individuals with different ages. The links on the static contact layer were assumed constant, whereas the links on the temporal contact layer were updated every day. Population of 100,000 individuals is synthesized following the age distribution from the census data of Shanghai, China [2] and connected on the static contact layer and the temporal contact layer. Configuration model was used to construct both the static contact layer and the temporal contact layer [3].

Following is the computational steps to construct the static contact layer:

- Step 1: Initialize nodes. Age group is assigned to each node, and the degree of a node in age group $\alpha$ is determined following the distribution: $P_{static}^{\alpha}(k)$. A node with degree $k$ has $k$ stubs and the stubs of all nodes are stored in the stub array.
- Step 2: Construct a link. Randomly choose one stub $i$ from stub array, then determine the age group of the other end of the link following the distribution: $P_{static}^{\alpha}$($\beta$). Then, randomly choose a stub from the stub array with age group $\beta$. If the two stubs chosen above are not from the same node or already connected, they are connected by a link and these two stubs are deleted from the stub array.
- Step 3: Repeat Step 2. Step 2 is repeated until all the stubs are connected or the remained stubs are from the same node or already connected.

Following is the computational steps to construct the temporal contact layer:

- Step 1: Initialize nodes. Whether nodes have random contacts or not is determined following the distribution: $P_{temp}^{\alpha}$, and the nodes are stored in the node array.
- Step 2: Construct a group. Randomly choose a node $i$ from the node array (and removed from the node array). Then, the size of the group (the node $i$ is not counted) is determined following the distribution: $P_{temp}(k)$. The age group of $k$ members in the group is determined following the distribution: $P_{temp}^{\alpha}(\beta)$. A node of age group $\beta$ is randomly selected from the node array (and removed from the node array), and repeat this process $k$ times to form the group. All nodes in the same groups are connected equally (i.e., homogeneous mixing).
- Step 3: $P_{temp}^{\alpha}($Repeat Steps 1 and 2. Step 1 and Step 2 are repeated until all nodes in the node array assigned to any of the groups.

The contact matrices for both the static and temporal contact layers are shown in **S7** and **S8 Fig**.

## SARS-CoV-2 Transmission model

**Modelling of transmission**

The SARS-CoV-2 transmission is simulated according to an SLIR (susceptible, latent, infectious, removed) scheme, where infectious individuals were further divided into pre-symptomatic (P), symptomatic (S), and asymptomatic (A) individuals (**S9 Fig**). In the model, the SARS-CoV-2 transmission occurs between susceptible and infectious individuals connected on one of the two layers (the static contact layer $s$ and the temporal contact layer $r$). At Day $t$, the probability that a susceptible individual $i$ gets infected through a contact with an infectious individual $j$ on layer $l$ is modeled as follows:

$p_{\left[ j\to i \right]}(t)$=${\lambda w}_{l}\delta\left( \alpha_{i} \right)\chi\left( m_{j} \right)h_{j}.$

Thus, the probability of transmission is dependent on the type of contact (static or temporal), age of susceptible individuals, and disease conditions ($P, I, A$). The values and description of the parameters composing the probability of infection are summarized in **S1Table**. The transmission risk on the static contact layer was calculated as weighted average of those of household, extended family, and social contacts using the contact counts as weights.

**Modelling of disease progression**

Those in $S$ (susceptible) status move to $L$ (latent) status once they infected. Those in $L$ status move to either $P$ (pre-symptomatic) status or $A$ (asymptomatic) status once they acquire infectiousness. Those in $P$ status develop symptom and move to $I$ (symptomatic) status. Finally, those in $I$ and $A$ statuses lose infectiousness and move to $R$ (removed) status, in which they are fully immune or dead. The parameters for the disease progression were set following epidemiological studies and summarized in **S1 Table**.

## Calibration

The scaling parameter in the transmission probability, $\lambda$ was calibrated by running the simulation. The epidemic growth rate ($r$) is computed by fitting an exponential curve to the daily new cases over time at initial phase of the epidemic. The reproduction number, $R_{0}$ is computed from $r$ as follows [10]:

$$R_{0}=\frac{r}{\sum_{i=1}^{n} y_{t}\left( e^{-ra_{i-1}}-e^{-ra_{i}} \right)/(a_{i}-a_{i-1})}$$

where $a_{0}$,$a_{1}$, $a_{2}$, $a_{3}$,…, $a_{n}$, are the category bounds of the histogram of the generation time, $y_{1}$, $y_{2}$,…, $y_{n}$ are the corresponding relative frequencies where the observed generation time are within these bounds. The value of $\lambda$ was determined such that the computed $R_{0}$ matches the pre-set value.

## The Test-Trace-Isolate strategy

We implemented symptom-based testing, contact tracing, and case isolation (namely Test-Trace-Isolate strategy [TTI]) in the simulation. Following is the processes of TTI:

**Test and isolation for symptomatic individuals**

Once infected individuals develop symptoms (move from $P$ to $I$), multiple actions are taken for them in the following order:

1. Test. Those who develop symptoms are tested by RT-PCR with probability $P_{test}$. For those who are tested, sample collection is conduced $T_{sc}$ days after symptom onset. The test result is returned $T_{cr}$ days after the sample collection. The true positive rate (i.e., sensitivity) is dependent on the time of sample collection after symptom onset, $\tau$: $P_{pcr}(\tau)$.
2. Disconnection of links (isolation). Links on both layers remain until sample collection. Between sample collection and test result return, links on temporal contact layer are disconnected, as they avoid unnecessary activities. In the sensitivity analysis, we run simulation assuming (1) links on both layers are disconnected or (2) all links remain active while the test results are waited.
   - If the test result is negative (false negative), the disconnected links recover, as they go back to normal activity.
   - If the test result is positive (true positive), the links of infected individuals on both layers are disconnected (i.e., isolated) for 14 days.

**Contact trace, test, and isolation for traced individuals**

Once infection is confirmed by RT-PCR tests, contacts of the confirmed cases are traced through the links on both contact layers and multiple actions are taken for the connected individuals in the following order:

1. Trace. All individuals connected to the individuals with positive test results on the static contact layer are traced. On the temporal contact layer, those who are connected within $D_{trace}$ days before sample collection of primary cases are traced with a probability $P_{trace}$. The tracing process is assumed to be completed without delay, and the sample is immediately collected from the traced individuals.
2. Test. For those successfully traced, sample collection is immediately conducted. Note that the false-positive rate (i.e., specificity) is assumed 100%.
3. Disconnection of links (isolation). Links on both layers remain until sample collection. Between sample collection and test result return, links on temporal contact layer are disconnected, as they avoid unnecessary activities. In the sensitivity analysis, we run simulation assuming (1) links on both layers are disconnected or (2) all links remain active while the test results are waited.
   - If the test result is negative (false negative), the disconnected links recover, as they go back to normal activity.
   - If the test result is positive (true positive), the links of infected individuals on both layers remain disconnected (i.e., isolated) for 14 days.

Note that if the infection of the traced individuals is confirmed, further tracing is performed subsequently. See the parameter values relevant to the TTI strategy in **S2 Table**.

## Reactive distancing policy

In response to the COVID-19 pandemic, health authorities have recommended non-pharmaceutical interventions (NPIs). TTI is one of effective NPIs. The advantage of NPIs over pharmaceutical interventions (such as vaccines and antiviral therapies) is that they do not require development time and can thus be promptly adopted as well as their effectiveness in inhibiting transmission, at least for a short term.

Here we considered another NPI, distancing policy, in addition to TTI. Distancing policy, limiting social events and gathering, has also been accepted in many countries and regions to mitigate the risk of SARS-CoV-2 transmission especially at early phase of the pandemic.

Here we considered two possible distancing policies: 1. reactive social distancing, limiting unnecessary activities only (a fraction of contacts on temporal contact layer are disconnected), 2. reactive “all-level” distancing, limiting both unnecessary and necessary activities (a fraction of contacts on both contact layers are disconnected). Those policies are triggered when the cumulative number of detected cases exceeds the predefined threshold value. See **S3 Table** for the parameters for reactive distancing policies.

## Additional results

### Additional disease burden results for the baseline analysis

In addition to the disease burdens presented in **Fig 3** (cumulative infections and deaths), we computed other disease burdens (symptomatic infections, hospitalization, ICU requirement) and confirmed similar trend (**S10 Fig**).

### 7.2 Sensitivity analysis varying the time from sample collection to laboratory diagnosis.

In the main analysis, we found that the impact of the time from sample collection to laboratory diagnosis ($T_{cr}$) on the reduction of infections (or effectiveness of TTI strategy) is influenced by the transmissibility ($R_{0}$) (**Fig 4D**). Longer $T_{cr}$ increases the infections under low transmissibility scenario (such as $R_{0}$=1.3); however, it decreases the disease infections under high transmissibility scenario (such as $R_{0}$=2.5).

Here we further investigated the impact of $T_{cr}$ on the average number of secondary infections (per a primary case) while waiting for the test results (**S11 Fig A**), the observed true positive rate (**S11 Fig B**), that for symptomatic individuals (**S11 Fig C**), and that for those recruited through contact tracing (**S11 Fig D**). Note that we only considered the first test, when multiple tests were performed. As we explained the mechanisms of the complex association between $T_{cr}$ and the reduction of infections under different $R_{0}$, the observed true positive rate, especially among those recruited through contact tracing was reduced by high $R_{0}$.

### 7.3 Sensitivity analysis varying infectiousness of asymptomatic individuals relative to symptomatic individuals

We assumed that the infectiousness of asymptomatic individuals is the same as that of symptomatic individuals in the main analysis. Here we run sensitivity analyses assuming the infectiousness of asymptomatic individuals is 50% of that of symptomatic individuals. The lower infectiousness of asymptomatic individuals yielded slightly higher effectiveness of TTI on reducing infections (**S12 Fig A**) and deaths (**S12 Fig B**).

### 7.4 Sensitivity analysis varying the number of initially infected individuals

We assumed 5 initially infected individuals in the main analysis. Here we run sensitivity analysis changing the number to 1 or 10. The effectiveness of TTI in reducing infections (**S13 Fig A**) and deaths (**S13 Fig B**) was not influenced by the initial number of infections.

### 7.5 Sensitivity analysis varying the probability to test a symptomatic individual

Probability to test a symptomatic individual, $P_{test}$, was assumed 0.8 in the main analysis. Here we run sensitivity analysis changing $P_{test}$ to 0.6 (low) or 1.0 (high). Higher $P_{test}$ was associated with higher effectiveness of TTI on reducing infections (**S14 Fig A**) and deaths (**S14 Fig B**).

### 7.6 Sensitivity analysis varying the number of detected cases that trigger the reactive distancing policy

In the main text, we assumed reactive distancing policy is triggered when the cumulative number of detected cases (the reactive threshold value) reaches 50. Here we explored scenarios where the reactive threshold values are 1 or 100 cases. The impact of the reactive threshold values on the effectiveness of TTI on reducing infections (**S15 Fig A** for reactive social distancing and **S15 Fig C** for reactive all-level distancing) and deaths were negligible (**S15 Fig B** for reactive social distancing and **S15 Fig D** for reactive all-level distancing). This is most likely because the cases increase exponentially at the early period of the epidemic, and the distancing policy is triggered almost at the same day under the scenarios considered here.

### 7.7 Daily new infections and new deaths

From the **S16 Fig** , we can see that in most scenarios, the epidemic ends within 1 year. As shown in the **S16 Fig A** and **S16 Fig C**, compared to unmitigated scenario, when TTI alone is implemented, the peak of new infections is markedly reduced. When alternative reactive distancing policy is implemented, the epidemic curve has been markedly flatted.

## Reference

1. Zhang J, Klepac P, Read JM, Rosello A, Wang X, Lai S, et al. Patterns of human social contact and contact with animals in Shanghai, China. Scientific Reports. 2019;9(1):15141. <https://doi.org/10.1038/s41598-019-51609-8> PMID: 31641189
2. Yang J, Marziano V, Deng X, Guzzetta G, Zhang J, Trentini F, et al. Despite vaccination, China needs non-pharmaceutical interventions to prevent widespread outbreaks of COVID-19 in 2021. Nature Human Behavior. 2021;5(8):1009-20. <https://doi.org/10.1038/s41562-021-01155-z> PMID: 34158650
3. Aleta A, Ferraz de Arruda G, Moreno Y. Data-driven contact structures: From homogeneous mixing to multilayer networks. PLoS Computational Biology. 2020;16(7):e1008035. <https://doi.org/10.1371/journal.pcbi.1008035> PMID: 32673307
4. Sun K, Wang W, Gao L, Wang Y, Luo K, Ren L, et al. Transmission heterogeneities, kinetics, and controllability of SARS-CoV-2. Science. 2021;371(6526). <https://doi.org/10.1126/science.abe2424> PMID: 33234698
5. Hu S, Wang W, Wang Y, Litvinova M, Luo K, Ren L, et al. Infectivity, susceptibility, and risk factors associated with SARS-CoV-2 transmission under intensive contact tracing in Hunan, China. Nature Communications. 2021;12(1):1533. <https://doi.org/10.1038/s41467-021-21710-6> PMID: 33750783
6. Aleta A, Martin-Corral D, Pastore YPA, Ajelli M, Litvinova M, Chinazzi M, et al. Modelling the impact of testing, contact tracing and household quarantine on second waves of COVID-19. Nature Human Behaviour. 2020;4(9):964-71. <https://doi.org/10.1038/s41562-020-0931-9> PMID: 32759985
7. Poletti P, Tirani M, Cereda D, Trentini F, Guzzetta G, Sabatino G, et al. Association of Age With Likelihood of Developing Symptoms and Critical Disease Among Close Contacts Exposed to Patients With Confirmed SARS-CoV-2 Infection in Italy. Jama Netw Open. 2021;4(3). <https://doi.org/10.1001/jamanetworkopen.2021.1085> PMID: 33688964
8. Zardini A, Galli M, Tirani M, Cereda D, Manica M, Trentini F, et al. A quantitative assessment of epidemiological parameters required to investigate COVID-19 burden. Epidemics. 2021;37:100530. <https://doi.org/10.1016/j.epidem.2021.100530> PMID: 34826786
9. Poletti P, Tirani M, Cereda D, Trentini F, Guzzetta G, Marziano V, et al. Age-specific SARS-CoV-2 infection fatality ratio and associated risk factors, Italy, February to April 2020. Euro Surveillance. 2020;25(31). <https://doi.org/10.2807/1560-7917.ES.2020.25.31.2001383> PMID: 32762797
10. Wallinga J, Lipsitch M. How generation intervals shape the relationship between growth rates and reproductive numbers. Proceedings of the Royal Society B: Biological Sciences. 2007;274(1609):599-604. <https://doi.org/10.1098/rspb.2006.3754> PMID: 17476782
11. Xiao AT, Tong YX, Gao C, Zhu L, Zhang YJ, Zhang S. Dynamic profile of RT-PCR findings from 301 COVID-19 patients in Wuhan, China: A descriptive study. Journal of Clinical Virology. 2020;127:104346. <https://doi.org/10.1016/j.jcv.2020.104346> PMID: 32361324
12. National Health Commission of People's Republic of China, Protocol of prevention and control for COVID-19 (8th edition) (National Health Commission of People's Republic of China, 2021); <http://www.gov.cn/xinwen/2021-05/14/content_5606469.htm>
