# Supplementary figures and images for: Evaluating the impact of test-trace-isolate for COVID-19 management and alternative strategies
Source: PLoS Comput Biol. 2023 Sep 1;19(9):e1011423. doi: 10.1371/journal.pcbi.1011423 (PMC10501547; doi:10.1371/journal.pcbi.1011423)

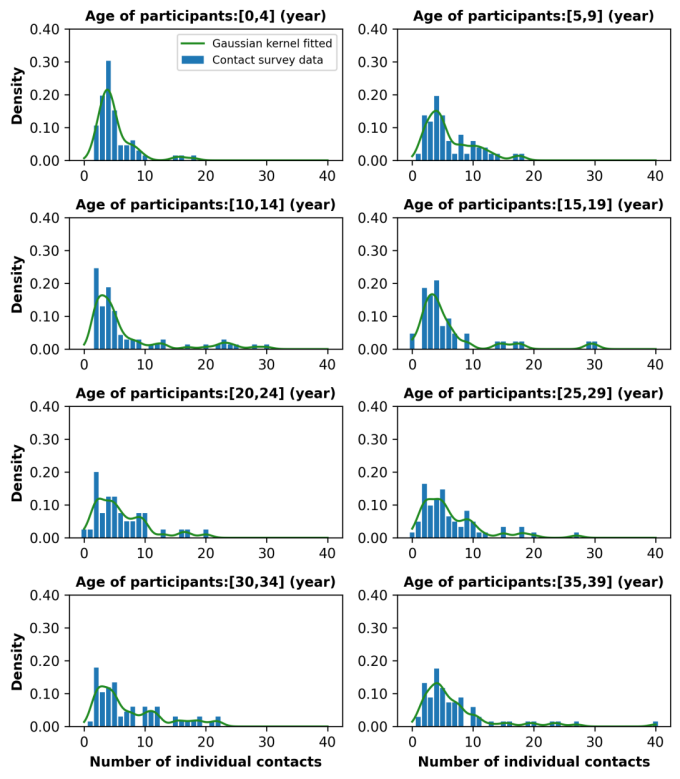

Supplement: S1 Fig — (TIF) [file pcbi.1011423.s002.tif]

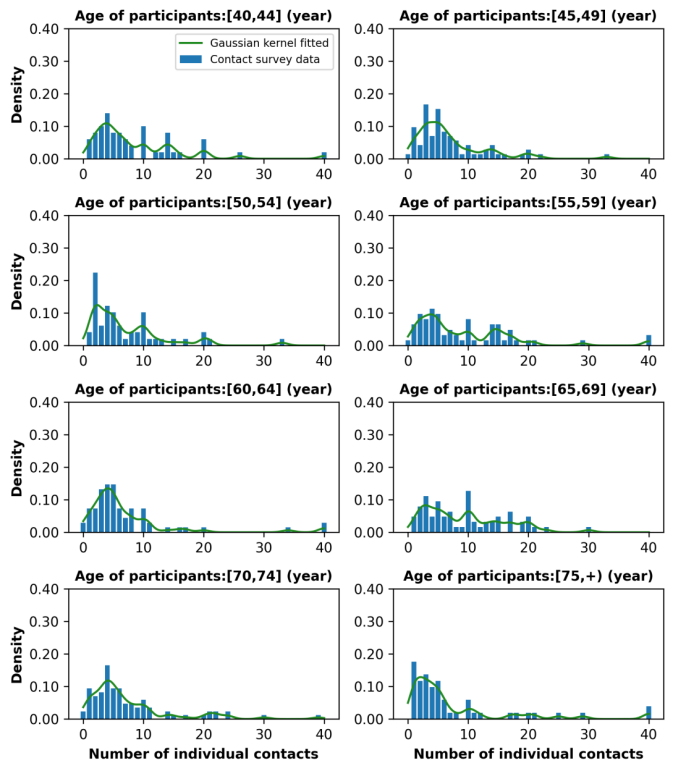

Supplement: S2 Fig — (TIF) [file pcbi.1011423.s003.tif]

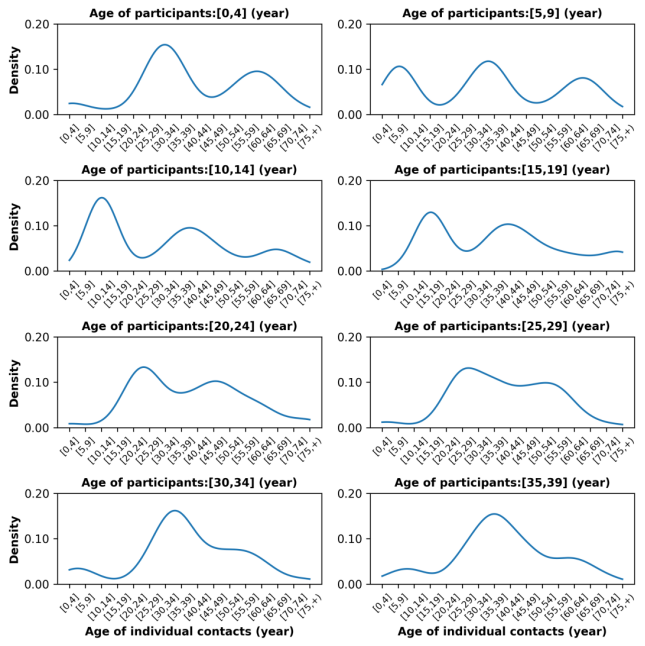

Supplement: S3 Fig — (TIF) [file pcbi.1011423.s004.tif]

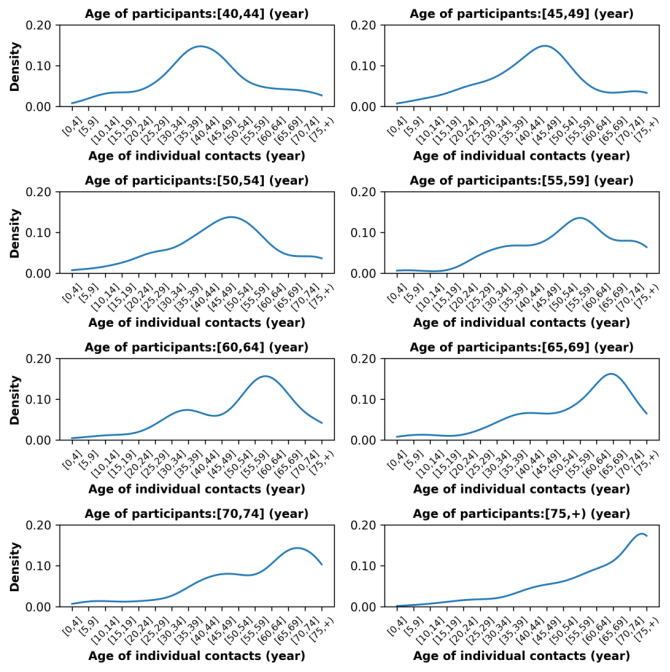

Supplement: S4 Fig — (TIF) [file pcbi.1011423.s005.tif]

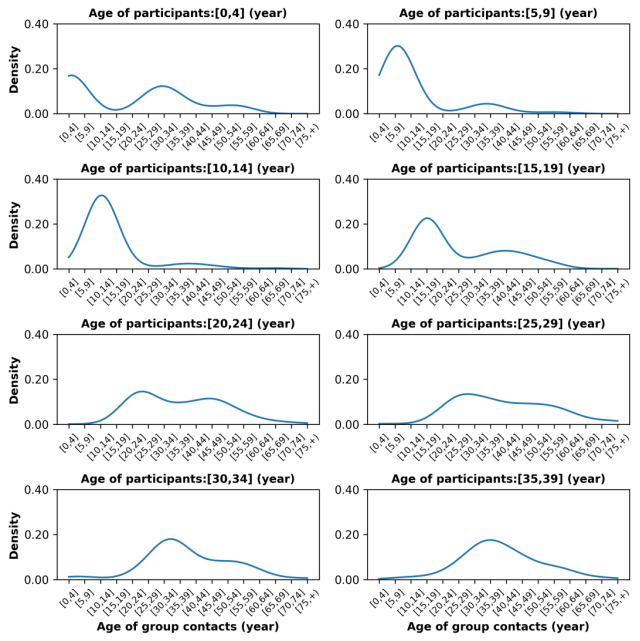

Supplement: S5 Fig — (TIF) [file pcbi.1011423.s006.tif]

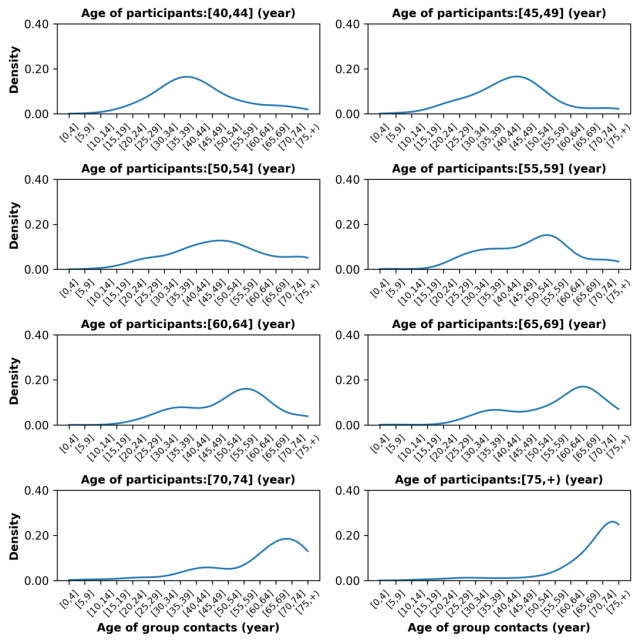

Supplement: S6 Fig — (TIF) [file pcbi.1011423.s007.tif]

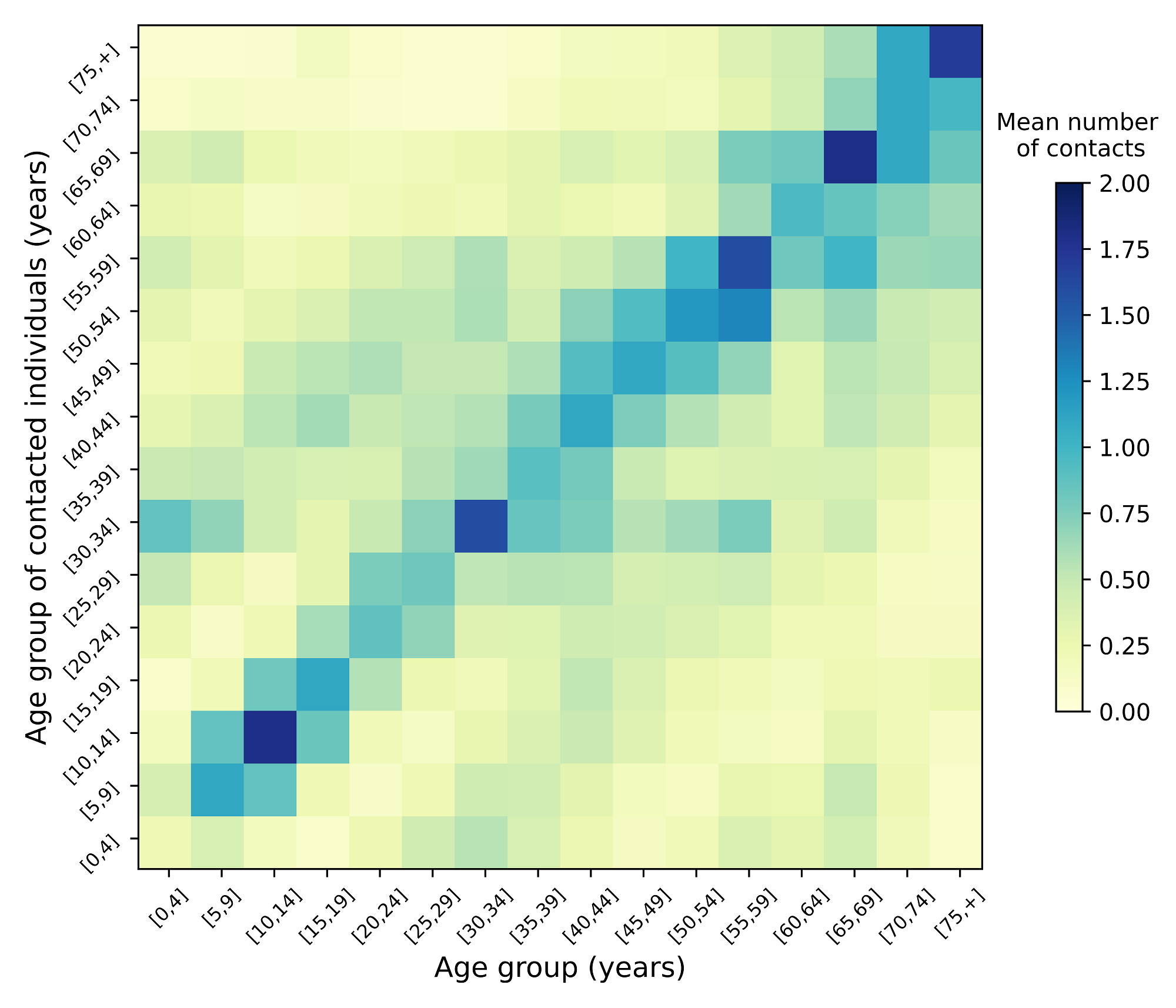

Supplement: S7 Fig — Colors represent the mean number of contacts between an individual in given age group with individuals with any age group. (TIF) [file pcbi.1011423.s008.tif]

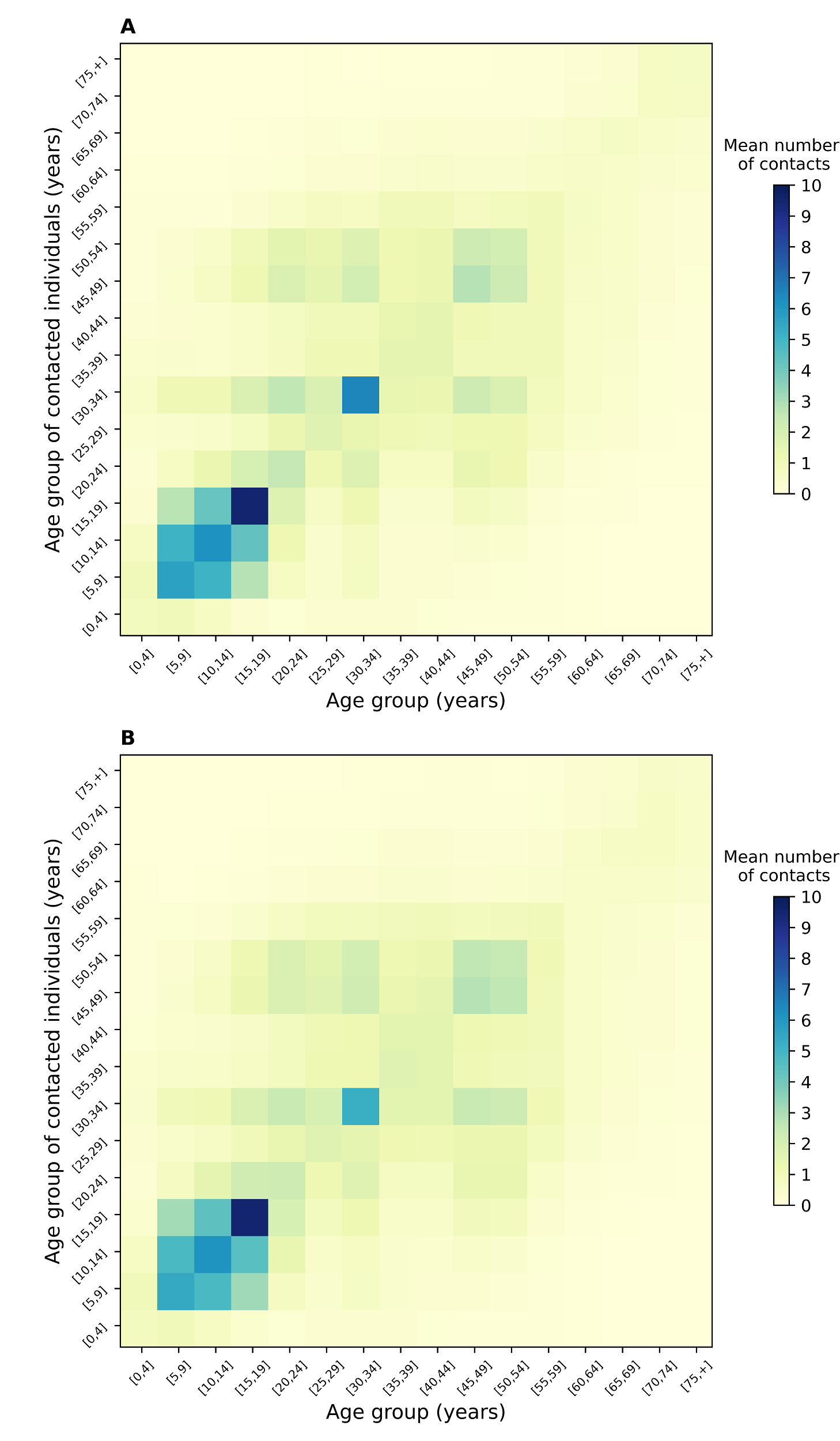

Supplement: S8 Fig — The panels show two realizations of the temporal contact layers for two randomly selected day of a simulation. Colors represent the mean number of contacts between an individual in given age group with individuals with any age group. (TIF) [file pcbi.1011423.s009.tif]

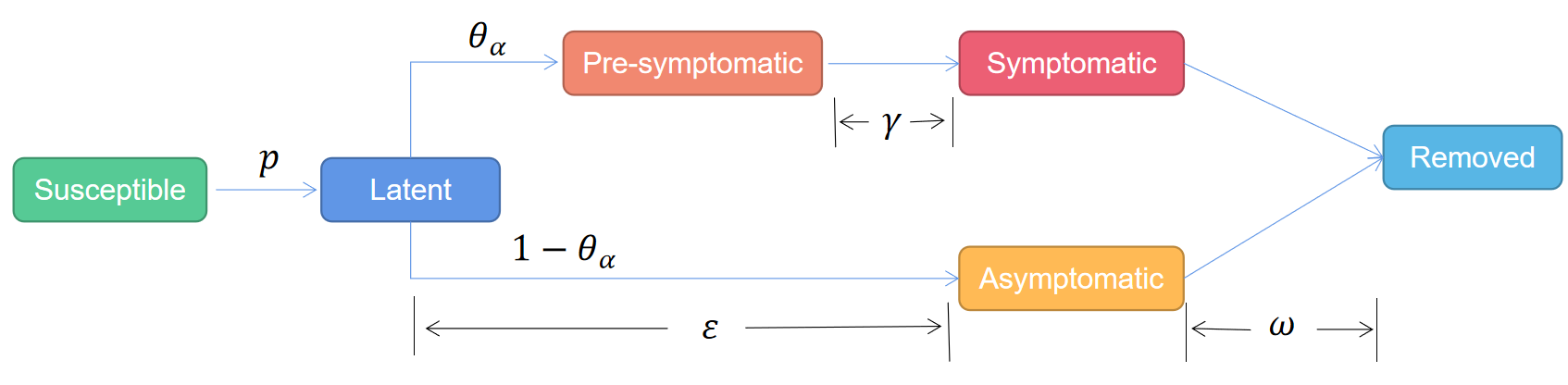

Supplement: S9 Fig — The detailed description of symbols in the figure referred to S1 Table. (TIF) [file pcbi.1011423.s010.tif]

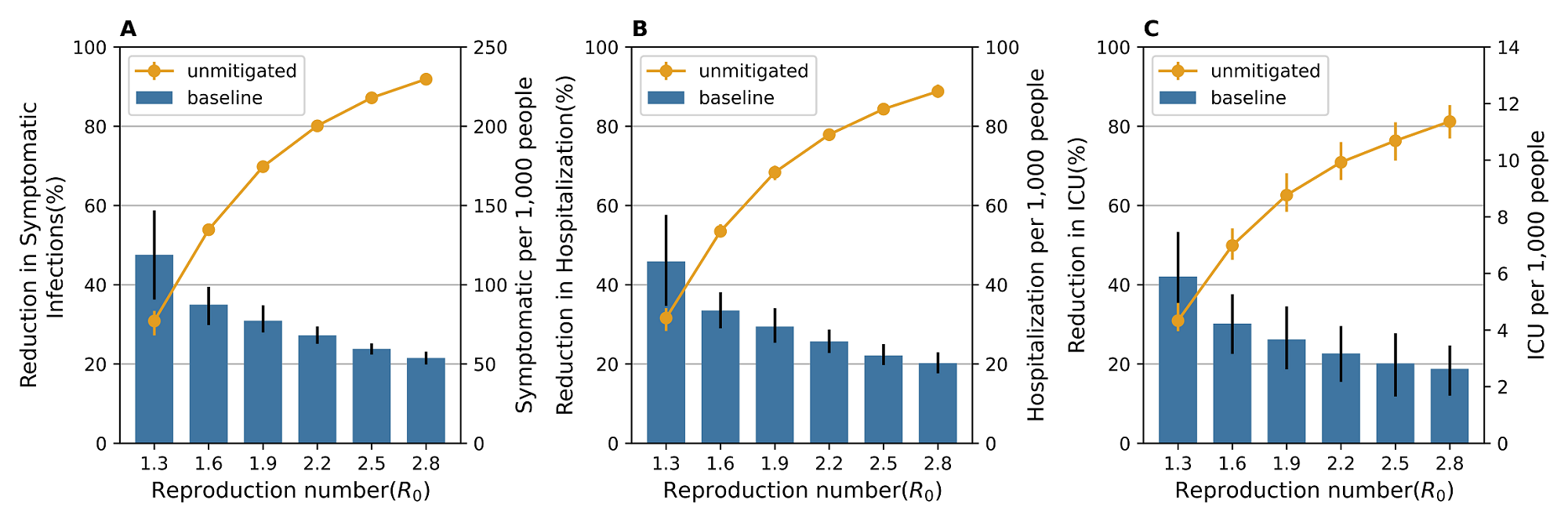

Supplement: S10 Fig — A. The number of symptomatic infections per 1,000 people without the TTI strategy (orange dots) and the reduction by the baseline TTI strategy (blue bars). Reproduction numbers (R0) are varied. The vertical error bars indicate the 95% CI. B. The same as A, but using the number of hospitalization as the disease burden. C. The same as A, but using the number of ICU patients as the disease burden. (TIF) [file pcbi.1011423.s011.tif]

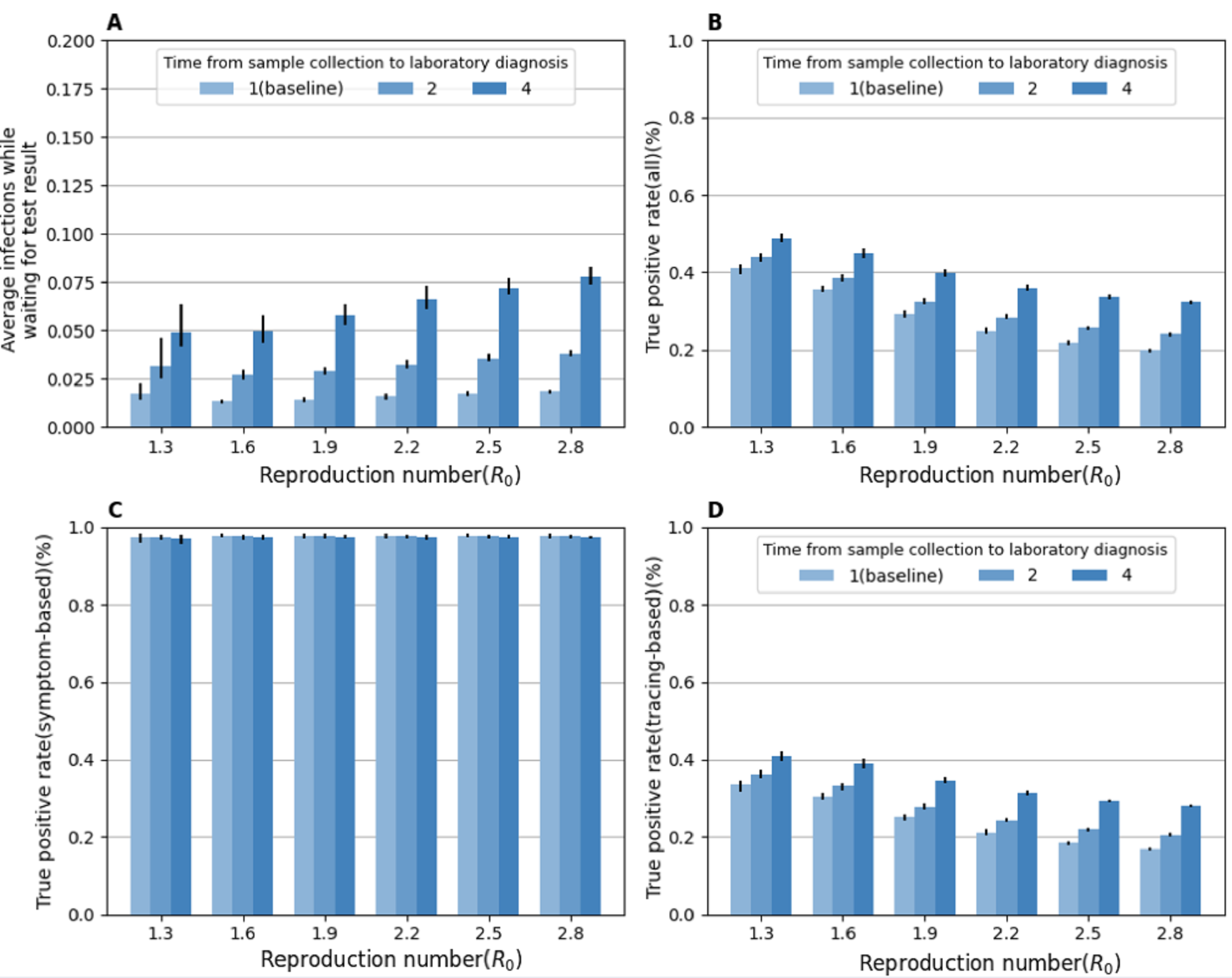

Supplement: S11 Fig — A. Average number of secondary infection (per a primary case) while waiting for the test results. B. observed true positive rate. C. as in B, but only for the symptomatic individuals. D. as in B, but only for those recruited through contact tracing. (TIF) [file pcbi.1011423.s012.tif]

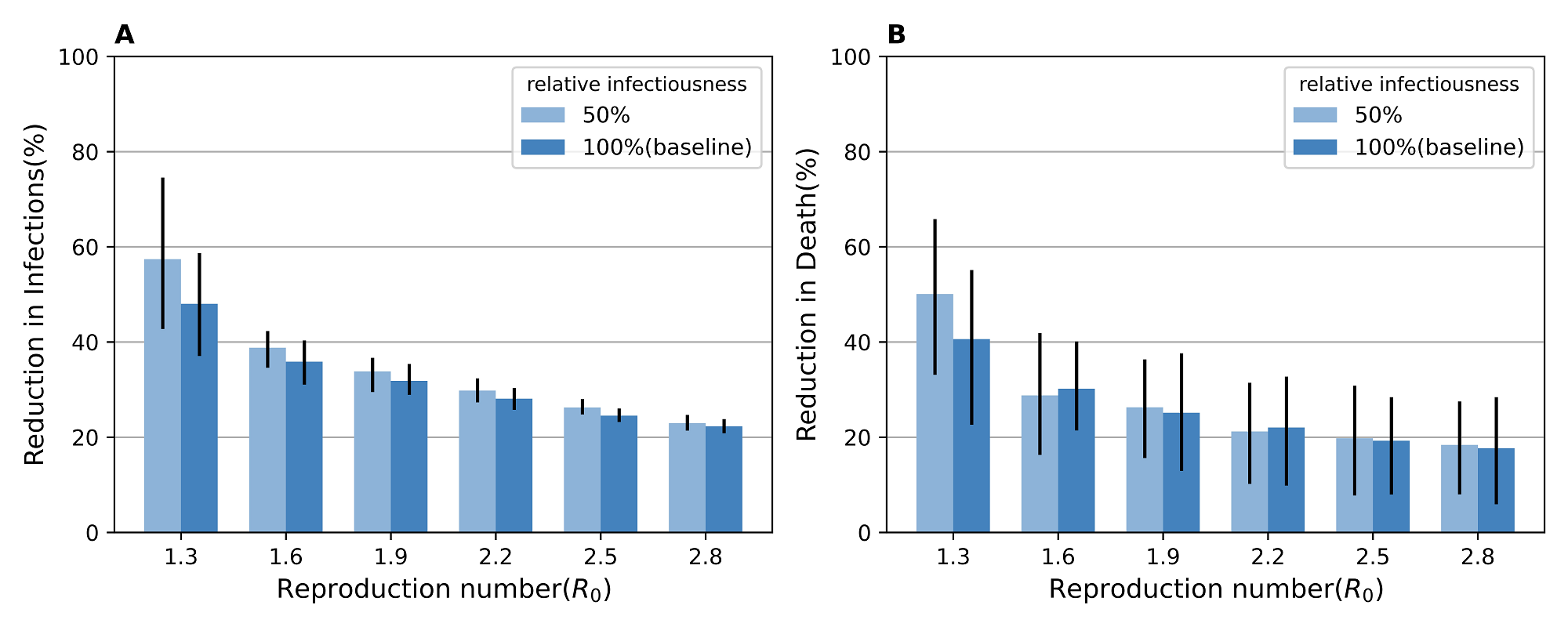

Supplement: S12 Fig — A. Reduction in the cumulative infections under different reproduction number and the relative infectiousness of asymptomatic cases. The vertical error bars indicate the 95% CI. B. as in A, but reduction in deaths. (TIF) [file pcbi.1011423.s013.tif]

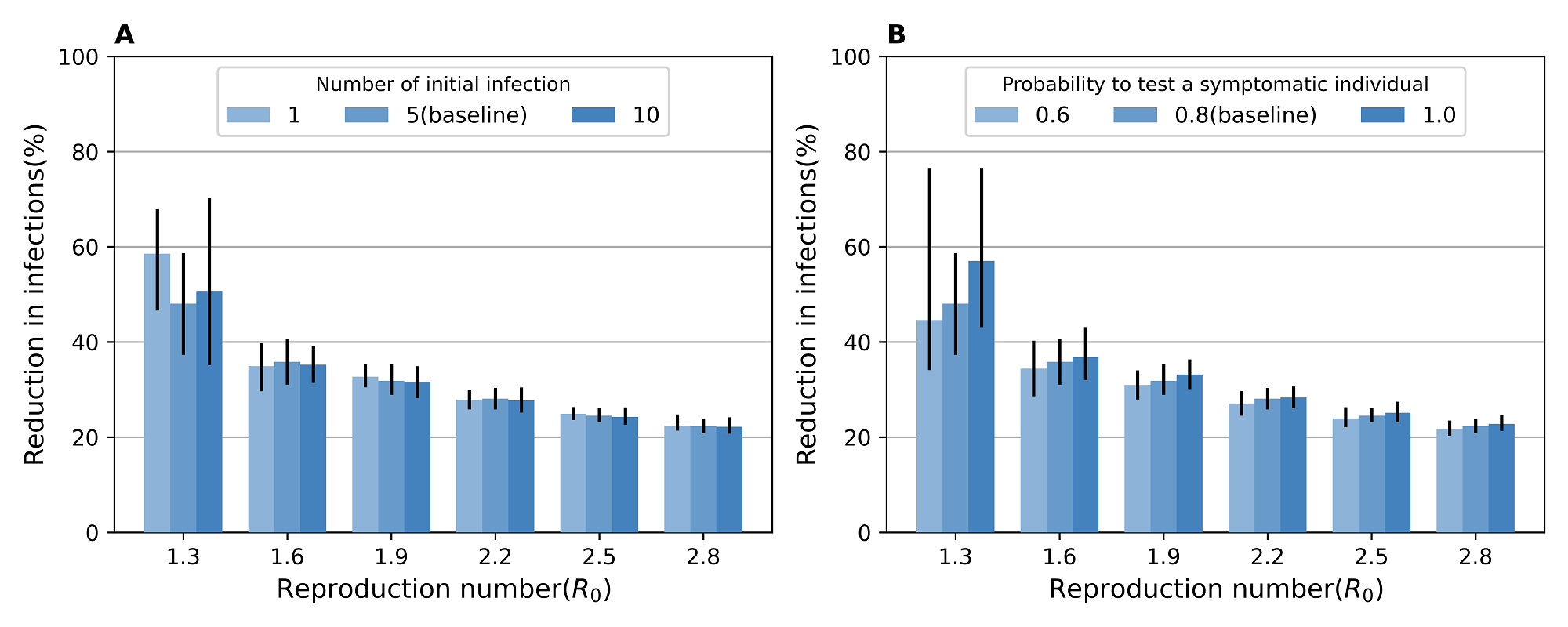

Supplement: S13 Fig — A. Reduction in the cumulative infections under different reproduction number and the initial number of cases. The vertical error bars indicate the 95% CI. B. as in A, but reduction in deaths. (TIF) [file pcbi.1011423.s014.tif]

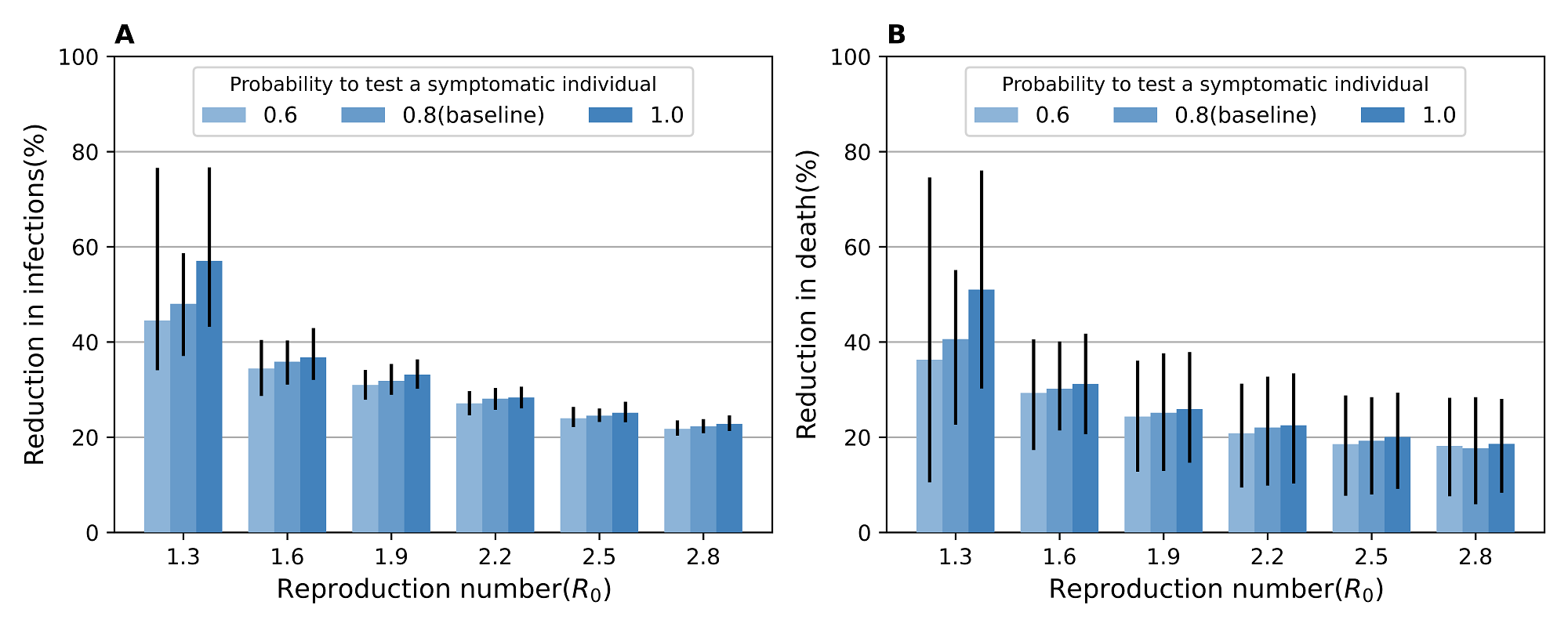

Supplement: S14 Fig — A. Reduction in the cumulative infections under different reproduction number and the probability to test a symptomatic individual. The vertical error bars indicate the 95% CI. B. as in A, but reduction in deaths. (TIF) [file pcbi.1011423.s015.tif]

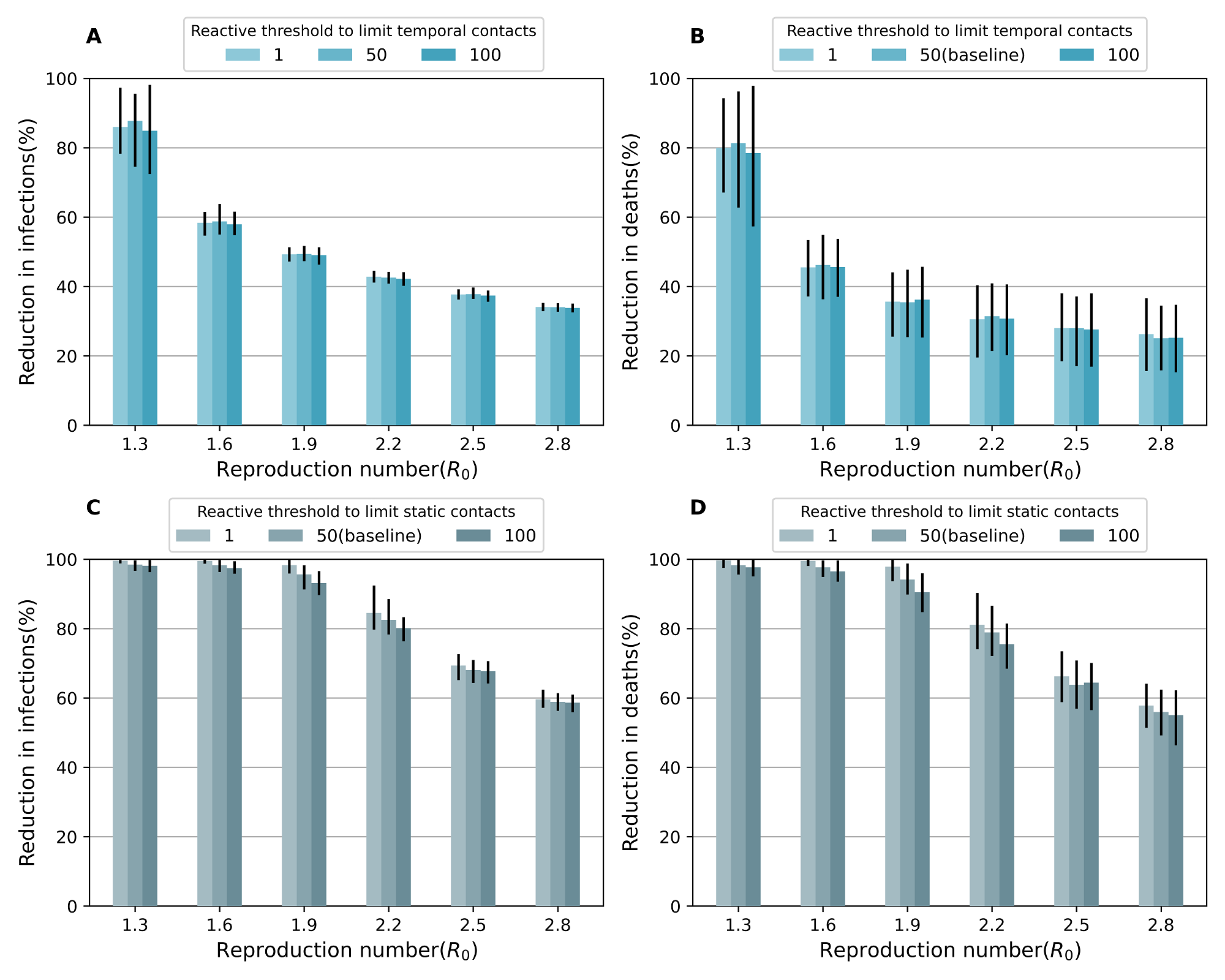

Supplement: S15 Fig — A. Reduction in the cumulative infections under different reproduction number and the reactive threshold values for the reactive social distancing. The vertical error bars indicate the 95% CI. B. as in A, but reduction in deaths. C. as in A, but for the reactive all-level distancing. D. as in C, but reduction in deaths. (TIF) [file pcbi.1011423.s016.tif]

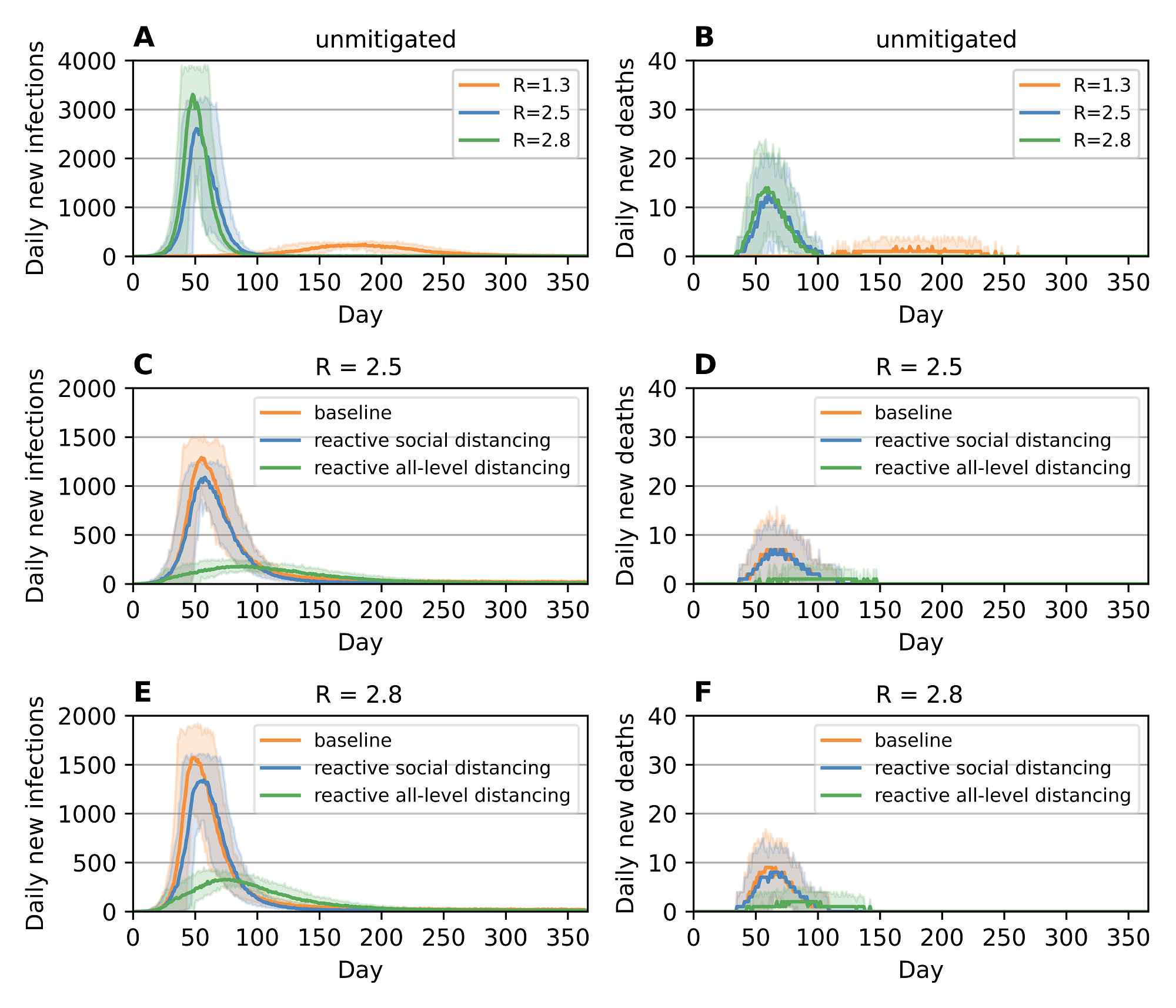

Supplement: S16 Fig — A. and B. for the daily new infections and daily new deaths under the unmitigated scenario (without TTI) with reproduction number of R0 = 1.3, 2.5 and 2.8, the shadows indicate the 95% CI; C. and D. for baseline where TTI is implemented under the scenario that the reactive social distancing is triggered when the cumulative number of detected cases exceeds 50, specifically, 50% of contacts on the temporal layer are disconnected, and under the scenario that the reactive all-level distancing is triggered when the cumulative number of detected cases exceeds 50, specifically, 50% of contacts on the temporal layer are disconnected and 30% of contacts on the static contact layer are disconnected. R0 = 2.5. E. and F. as in C. and D., but R0 = 2.8. (TIF) [file pcbi.1011423.s017.tif]
