# Supplementary material for: Evaluating the impact of test-trace-isolate for COVID-19 management and alternative strategies
Source: PLoS Comput Biol. 2023 Sep 1;19(9):e1011423. doi: 10.1371/journal.pcbi.1011423 (PMC10501547; doi:10.1371/journal.pcbi.1011423)
Supplement: S1 Table — (DOCX) [file pcbi.1011423.s018.docx]

**Table S1. Parameters for SARS-Cov-2 transmission model and disease burden.**

| **Transmission parameters** | | | | |
| --- | --- | --- | --- | --- |
| **Notation** | **Description** | **Value (or range)** | **Value(s) for sensitivity analysis** | **Reference** |
| $\lambda$ | Scale parameter | Determined by $R$  (See “4. Calibration”) | - | - |
| $w_{l}$ | Relative transmission risk of a contact on the contact layer $l$ compared with that on the temporal contact layer $r$: $l \in\{s, r\}$ | $w_{r}$=1.00 (by definition);  $w_{s}$=2.84 | - | [4] |
| $h_{j}$ | Transmission heterogeneity of an individual $j$ | Gamma distribution  (shape=0.3, scale=3.3) | - | [4] |
| $\delta\left( \alpha_{i} \right)$ | Relative susceptibility to SARS-CoV-2 infection of individual $i$ in age group $\alpha$ | $\delta\left( \alpha\right)$= 0.58, $\alpha<15$;  $\delta\left( \alpha\right)$= 1.00, $15\leq\alpha<65$;  $\delta\left( \alpha\right)$= 1.65, $65\leq\alpha$ | - | [5] |
| $\chi\left( m_{j} \right)$ | Relative infectiousness of an individual $j$ in the infectious status $m$ compared with that of pre-symptomatic ($P$) or symptomatic ($I$) individuals: $m\in\{P,I,A\}$ | $\chi\left( P \right)=\chi\left( I \right)=1.0$ (by definition);  $\chi\left( A \right)=1.0$ | $\chi\left( A \right)=0.5$ | [5] |
| **Disease progression parameters** | | | | |
| **Notation** | **Description** | **Value (or range)** | **Value(s) for sensitivity analysis** | **Reference** |
| $T_{g}$ | Generation time (time interval between infections of primary and secondary cases) | 7.0 days (IQR: 3.6-11.3) | - | [4] |
| $R_{0}$ | Reproduction number | 2.5 | 1.3, 1.6, 1.9, 2.2, 2.8 | [5] |
| $\gamma$ | Pre-symptomatic period (period that an infected individual stays in $\boldsymbol{P}$ status) | 2.0 days | - | [5] |
| $\varepsilon$ | Incubation period (period that an infected individual stays in $\boldsymbol{E}$; time interval from infection to symptom development) | Gamma distribution  with mean 6.3 (days)  (shape=2.08，scale=0.33) | - | [5] |
| $\omega$ | Recovery period (period that an infected individual stays in $\boldsymbol{I}$ or $\boldsymbol{A}$) | Exponential distribution. The mean value was estimated to match the mean generation time (7.0 days) | - | [6] |
| $\theta_{\alpha}$ | Symptomatic ratio (probability that an infected individual in age group $\alpha$ develops respiratory symptoms and/or fever) | $\theta_{\alpha}$=0.181, $\alpha<20$;  $\theta_{\alpha}$=0.224, $20\leq\alpha<40$;  $\theta_{\alpha}$=0.305, $40\leq\alpha<60$;  $\theta_{\alpha}$=0.355, $60\leq\alpha<80$;  $\theta_{\alpha}$=0.646, $80\leq\alpha$ | - | [7] |
| **Disease burden parameters** | | | | |
| **Notation** | **Description** | **Value (or range)** | **Value(s) for sensitivity analysis** | **Reference** |
| $p_{\alpha}$ | Hospitalization risk (probability that an infected individual in age group $\alpha$ is hospitalized) | $p_{\alpha}$=0.025, $\alpha<15$;  $p_{\alpha}$=0.074, $15\leq\alpha<20$;  $p_{\alpha}$=0.053, $20\leq\alpha<40$;  $p_{\alpha}$=0.130, $40\leq\alpha<60$;  $p_{\alpha}$=0.172, $60\leq\alpha<70$;  $p_{\alpha}$=0.275, $70\leq\alpha<80$;  $p_{\alpha}$=0.430, $80\leq\alpha$ | - | [8] |
| $q_{\alpha}$ | Critical illness risk (probability that an infected individual in age group $\alpha$ develop critical illness) | $q_{\alpha}$=0.000, $\alpha<20$;  $q_{\alpha}$=0.004, $20\leq\alpha<40$;  $q_{\alpha}$=0.009, $40\leq\alpha<60$;  $q_{\alpha}$=0.026, $60\leq\alpha<70$;  $q_{\alpha}$=0.072, $70\leq\alpha<80$;  $q_{\alpha}$=0.184, $80\leq\alpha$ | - | [7] |
| $d_{\alpha}$ | Infection fatality risk of individuals in age group $\alpha$ | $d_{\alpha}$=0.000, $\alpha<50$;  $d_{\alpha}$=0.004, $50\leq\alpha<60$;  $d_{\alpha}$=0.090, $60\leq\alpha<70$;  $d_{\alpha}$=0.056, $70\leq\alpha<80$;  $d_{\alpha}$=0.081, $80\leq\alpha$ | - | [9] |
