# Supplementary material for: Evaluating the impact of test-trace-isolate for COVID-19 management and alternative strategies
Source: PLoS Comput Biol. 2023 Sep 1;19(9):e1011423. doi: 10.1371/journal.pcbi.1011423 (PMC10501547; doi:10.1371/journal.pcbi.1011423)
Supplement: S2 Table — (DOCX) [file pcbi.1011423.s019.docx]

**Table S2. Parameters for TTI**

| **Testing parameters** | | | | |
| --- | --- | --- | --- | --- |
| **Notation** | **Description** | **Value (or range)** | **Value(s) for sensitivity analysis** | **Reference** |
| $P_{test}$ | Probability to test a symptomatic individual | 80% | 60%, 100% | - |
| $P_{pcr}(\tau)$ | True positive rate (sensitivity) as a function of the time from symptom onset to sample collection ($\tau$ days) | $P_{pcr}(\tau)$=0.979, $0<\tau\leq7$;  $P_{pcr}(\tau)$=0.686, $7<\tau\leq14$;  $P_{pcr}(\tau)$=0.363, $14<\tau\leq21$;  $P_{pcr}(\tau)$=0.300, $21<\tau\leq28$;  $P_{pcr}(\tau)$=0.000, $28<\tau$; | - | [11] |
| $T_{sc}$ | Mean delay between symptom onset and sample collection (days) | $T_{sc}\sim Pois(1)$ | $T_{sc}\sim Pois\left( 1 \right)$ or $Pois\left( 4 \right)$ | - |
| $T_{cr}$ | Time between sample collection and laboratory diagnosis (days) | $T_{cr}\sim Pois(1)$ | $T_{cr}\sim Pois\left( 2 \right)$ or $Pois\left( 4 \right)$ | - |
| **Contact trace and isolation parameters** | | | | |
| **Notation** | **Description** | **Value (or range)** | **Value(s) for sensitivity analysis** | **Reference** |
| $D_{trace}$ | Days to trace contacts on the temporal contact layer before sample collection of the primary cases | 4 days | 2, 6 | - |
| $P_{trace}$ | Probability that contacts on the temporal contact layer to be successfully traced | 50% | 30%, 70% | - |
| $T_{iso}$ | Duration of isolation (days) | 14 days | - | [12] |
