# Supplementary material for: Evaluating the impact of test-trace-isolate for COVID-19 management and alternative strategies
Source: PLoS Comput Biol. 2023 Sep 1;19(9):e1011423. doi: 10.1371/journal.pcbi.1011423 (PMC10501547; doi:10.1371/journal.pcbi.1011423)
Supplement: S3 Table — (DOCX) [file pcbi.1011423.s020.docx]

**Table S3. Parameters for reactive distancing policies**

| **Reactive social distancing** | | | | |
| --- | --- | --- | --- | --- |
| **Notation** | **Description** | **Value (or range)** | **Value(s) for sensitivity analysis** | **Reference** |
| $P_{social}$ | Proportion of contacts in the temporal contact layer to be disconnected | 50% | 25%, 75% | - |
| $T_{social}$ | Number of detected cases to trigger the reactive social distancing | 50 | 1, 100 | - |
| **Reactive all-level distancing** | | | | |
| $P_{static}$ | Proportion of contacts on the static contact layer to be limited | 30% | 10%, 50% | - |
| $P_{social}$ | Proportion of contacts on the temporal contact layer to be disconnected | 50% | - | - |
| $T_{all}$ | Number of detected cases to trigger the reactive all-level distancing | 50 | 1, 100 | - |
