# Supplementary material for: Evaluating the impact of test-trace-isolate for COVID-19 management and alternative strategies
Source: PLoS Comput Biol. 2023 Sep 1;19(9):e1011423. doi: 10.1371/journal.pcbi.1011423 (PMC10501547; doi:10.1371/journal.pcbi.1011423)
Supplement: Attachment — Submitted filename: response-letter.docx [file pcbi.1011423.s021.docx]

Dear Editor,

Please find enclosed the revised version of our manuscript “Evaluating the impact of Test-Trace-Isolate for COVID-19 management and alternative strategies” (Tracking#: PCOMPBIOL-D-23-00428) by Zhang et al.

We would like to thank you for giving us the opportunity to revise our manuscript. We are also truly grateful to the reviewers for their thorough assessment of our manuscript that was instrumental to greatly improving our work.

We enclose a point-by-point response to all comments and remarks of the reviewers, explaining the corresponding changes made in the manuscript.

We believe the manuscript has now considerably improved, and we dare to hope that it is now suitable for publication in PLoS Computational Biology.

Sincerely yours,

Keisuke Ejima and Quan-Hui Liu

**Detailed reply to Reviewers**

We are extremely grateful to the editor and the two reviewers for their thorough assessment of the manuscript. Their remarks and comments truly helped us to considerably improve our manuscript.

Reviewer #1:

The authors implement an agent-based model of SARS-CoV-2 disease spread where the contact patterns of agents are governed by two separate contact layers. The first represents close, regular contacts and the second represents casual, sporadic contacts. They incorporate the effects of test-trace-isolate strategies on contact rates within this modelling framework and investigate the consequences under different scenarios in terms of outcomes averted (total infections and deaths) and the cost of implementation (peak numbers of daily tests and simultaneously isolating individuals). The authors performed sensitivity analysis for several key parameters.

Overall, the study seems robust. However, I have a number of suggestions for improvement.

We would like to thank the reviewer for taking the time to review our manuscript and for the constructive comments. We are glad to hear that the reviewer think that our study is “robust”.

Major

Please include more details in the methods section. Unless the authors are limited by word counts, I suggest shifting some of the details from the methods supplement into the main text.

Thank you for this suggestion. We have shifted more details to the main text (**Methods** section). The revised parts include a description of the contact survey data and how they are used to construct the contact network.

It is not clear in the methods section that contact survey data are used to inform the contact rates. This is the first and only mention (line 144): “More detailed description of the survey data and network generation process is available in Supplementary Methods.” There is no description of the survey data in the methods so how can it be more detailed in the SM. Further, please provide a more detailed description of the contact survey design and resulting data structure. It is not sufficient to point to the reference. It must be at least be summarised here.

We apologize for the omission of these key details. As suggested, we have now added the following section in the **Methods** to describe the contact survey data (lines: 144-155):

“ **Contact survey data.** To reconstruct the network of contacts, we relied on data collected in a contact survey conducted between December 2017 and May 2018 in Shanghai, China [46]. Similar to the POLYMOD study [55], the survey collected basic sociodemographic information about each study participant (e.g., age, sex) and detailed information about each individual contacted by the participant during the 24 hours period before filling in the questionnaire, where a contact was defined as either a two-way conversation with three or more words in physical presence or a physical skin-to-skin contact. Information collected for each contact included age/age group, sex, contact location and duration, whether the contact was physical or not, and the social setting where the contact took place (e.g., school, workplace). The original contact survey data shows that households, schools, and workplaces were the social settings accounting for the majority of contacts but participants also reported large number of contacts (20+) that were difficult to record individually, like those related to social events (i.e., contacts in the general community). ”

In addition, in the section of **Static-temporal multiplex network model**, we now describe the steps we did to construct the static contact layer (lines: 166-170):

“ On the static contact layer, links are generated following age-dependent contact frequencies (**Fig. 1B**). Specifically, for each node, the number of links was sampled from the corresponding age-specific distribution. The static contact layer is assumed constant (thus the links are not re-generated or disappear during the simulation of an epidemic). ”

The first time the model is described as a stochastic agent-based model is in the Discussion. This type of detail must be included in the methods section.

Thank you for pointing this out. As suggested, we now have added this information at the beginning of the **Methods**, in the section “**Overview of transmission simulation with TTI”** (lines: 134-137):

“ To simulate SARS-CoV-2 transmission dynamics in a population, we developed a stochastic agent-based model [54] on a static-temporal multiplex network. The disease progression was modelled through a susceptible-exposed-infectious-removed (SEIR) scheme. The static-temporal multiplex network was composed of two layers: the static contact layer and the temporal contact layer. ”

Your network includes household contacts. Does your model incorporate household structure? If not, worth reflecting on the implications of this in the Discussion.

We apologize for the lack of clarity. Our model uses data on the number of contacts by age for different social settings, including the household. Therefore, the static layer of the contact network accounts for “realistic” household contacts. However, the model does not explicitly consider household structure. We fully agree with the reviewer that this is a limitation of our analysis, which needs to be discussed together with the other limitations. As such, we have added the following paragraph in the **Discussion** (lines: 407-411):

“ First, we grouped contacts that take place in the household, school, and workplace settings into a single layer (i.e., static contact). Therefore, our network does not explicitly replicate the structure of each household, school, and workplace. As such, our network cannot capture the saturation effect of household transmission observed in previous studies [67, 68], which may lead our model to slightly overestimate the infection attack rate. ”

Please include some plots of new infections (and deaths potentially too) over time. This is partly to confirm that the model behaviour is as expected, but also the authors state that the simulations are run for 1 year. Does the epidemic in all scenarios, including in the sensitivity analyses, “complete” within 1 year? It would also be useful to visualise for the reactive distancing scenarios how the epidemic trajectory changes with the addition of social restrictions.

Thank you for the suggestion. We now have added plots of daily number of new infections and daily number of new deaths over the simulation period (i.e., 1 year) in the **Supplementary Methods** (7. Additional results). **Figure S16** (reported in that section and appended below for reviewer’s convenience), we can see that simulated epidemic ends within 1 year.

**Fig. S16** **Daily new infections and new deaths.** **A.** and **B.** for the daily new infections and daily new deaths under the unmitigated scenario (without TTI) with reproduction number of $R_{0} = 1.3, 2.5 and 2.8$, the shadows indicate the 95% CI; **C.** and **D.** for baseline where TTI is implemented under the scenario that the reactive social distancing is triggered when the cumulative number of detected cases exceeds 50, specifically, 50% of contacts on the temporal layer are disconnected, and under the scenario that the reactive all-level distancing is triggered when the cumulative number of detected cases exceeds 50, specifically, 50% of contacts on the temporal layer are disconnected and 30% of contacts on the static contact layer are disconnected. $R_{0} =2.5$. **E.** and **F.** as in **C.** and **D.** , but $R_{0} =2.$8.

We would like to thank the reviewer once again as these new plots visually show very clearly the effect of TTI, especially in flattening the epidemic curve.

In the discussion, please discuss your work in the context of other studies. You started to do this (very briefly) in the Results (line 197) but this should be done more comprehensively in the discussion. E.g. how do your findings, including the relationship between different parameters and outcomes, compare to other modelling studies e.g., https://doi.org/10.1016/S1473-3099(20)30361-3

Thank you for suggesting this relevant article. As suggested, we have now contextualized our results in the **Discussion** and included the suggested paper (lines: 371-378):

“ When $R_{0}$ is 2.2, given that infectiousness onset almost coincides with symptom onset (infectiousness appears 0.51-0.77 days before symptom onset on average), Peak et al. [65] found that tracing contacts and quarantine or active monitoring individuals triggered by symptom presence have a certain potential to control the SARS-CoV-2 spreading. However, in situations where less resources are available for tracing, or the adherence to such interventions is low, the effectiveness of TTI could be low. For example, if less than 20% of the population follows the rule of self-reporting and isolation due to symptoms or tracing, large outbreaks are unlikely to be avoided even with scaled-up tracing and quick turnaround testing [28]. ”

You mention various limitations in the discussion. Please elaborate by speculating on how these limitations may have impacted your results.

As suggested, we have added an extensive discussion for each limitation we listed in the previous manuscript pointing out its possible impact on our results (lines: 419-428):

“ The assumption of full connectivity may increase the risk of pathogen transmission among individuals in the same group, as each susceptible individual in that group can be infected by any infectious member of the group. Third, our simulations assumed an instantaneous turnaround time for contact tracing, but contact tracing is often implemented through interviews conducted by an operator; thus, it may take several days to gather all information and identify contacts. Kretzschmar et al. ‘s study [69] have shown that the tracing delay has certain impact on the contact tracing effectiveness, although lower than the testing delay. Thus, the assumption of an instantaneous turnaround time for contact tracing may lead to a slightly overestimation of the effectiveness of TTI. ”

Minor

I suggest stating how your study fits into the epidemiological and policy context of the COVID-19 pandemic. The baseline R0 of 2.5 and other features (fully susceptible population??) of your analysis suggest that the study is focused on the era of ancestral SARS-CoV-2 virus (I.e. before the emergence of the Alpha variant and availability of vaccines).

We apologize for the lack of clarity, and we are grateful to the reviewer for pointing out that we were missing the epidemiological context for our analysis. The reviewer is correct. Our analysis refers to the early phase of the pandemic dominated by the ancestral SARS-CoV-2 lineage and a fully susceptible population. We have now included the following statement in the **Introduction** (lines: 123-128):

“ The transmission model was used to access the effectiveness of TTI on SARS-CoV-2 spread considering an epidemiological context consistent with the initial phase of the COVID-19 pandemic (i.e., ancestral SARS-CoV-2 lineages, fully susceptible population, no vaccines and antiviral therapies). We run epidemic simulations over one year for different values of the reproduction number and under different TTI strategies coupled with interventions restricting contacts. ”

I suggest deleting the first line of the introduction as this statement is not true.

We fully agree with the reviewer. As suggested, we have deleted the first line of the introduction.

Line 52. I suggest deleting “and the etiology was not clear” as this statement is not true. The etiology was clear in March 2020 when many countries first implemented lockdowns.

Thank you for pointing this out. As suggested, we have deleted this statement.

Line 52. “…due to the huge impacts of the lockdowns on the functioning of the society” AND the availability of other control measures such as vaccines.

Thank you for the suggestion - correction made.

Line 76. What do you mean by “silent transmission”?

We apologize for the lack of clarity. With silent transmission with refer to transmission from pre-symptomatic and asymptomatic individuals. We have revised the statement as follows (lines: 85-87):

“ Oran et al. [30] estimated that nearly 40% to 45% of SARS-CoV-2 infections occur during the pre-symptomatic and asymptomatic stages, which is crucial to the effectiveness of symptom-driven interventions [31]. ”

Line 79. Remove “etc” or explain what it is referring to.

Thank you for pointing this out – correction made.

Line 177. The first time SEIR is mentioned, it should be spelled out in full.

Thank you for pointing this out. We now have revised this statement.

For visualising the contact rates between age groups, it would be useful to see plots of the contact matrices for both the static and temporal contact layers.

Thank you for the suggestion. We now have added plots of the contact matrices by age for both the static and temporal contact layers. The new plots are reported in the **Supplementary Methods** (2. Static-Temporal Multiplex Network), and appended below for reviewer’s convenience.

**Fig. S7 Contact matrix representing the static contact layer.** Colors represent the mean number of contacts between an individual in given age group with individuals with any age group.

**Fig. S8 Contact matrix representing the temporal contact layer.** The panels show two realizations of the temporal contact layers for two randomly selected day of a simulation. Colors represent the mean number of contacts between an individual in given age group with individuals with any age group.

Line 145. The average numbers of contacts. Is this per day? 20+ seems quite high. Is this consistent with the survey data?

Thank you for pointing this out. The average numbers of contacts on the static contact layer and the temporal contact layer are 7.15 and 13.7 contacts per day, respectively, which add up to 20.85 contacts per day. This value is consistent with that in the original survey data (mean = 19.3 contacts per day). This estimate obtained in the Chinese contact survey we used is also consistent with other estimates obtained before the COVID-19 pandemic in European countries (e.g., 19.77 and 17.46 contacts per day on average were reported in Italy and Luxembourg, respectively – Mossong et al., Social contacts and mixing patterns relevant to the spread of infectious diseases. PLoS Medicine, 2008). This information has been added to the main text (lines: 176-180):

“ The average numbers of contacts for the static and temporal contact layers are 7.15 and 13.7, respectively, which add up to 20.85 contacts per day and is consistent with the original data (an average of 19.3 contacts per day) and other estimates obtained before the COVID-19 pandemic in European countries (e.g., 19.77 and 17.46 contacts per day on average were reported in Italy and Luxembourg, respectively) [55]. ”

How did you initialise the simulations? Is the population fully susceptible?

We apologize for the lack of detail. At the beginning of the simulation, we consider a fully susceptible population, expect for 5 (randomly selected) infected individuals. This information has been added to the **Methods** (lines: 210-213):

“ Simulations were initialized with a fully susceptible population (thus reflecting the situation at the start of the pandemic), except for 5 randomly selected latent individuals. For each analysis, results are based on 100 stochastic model realizations. ”

Line 218. Typographical error. Captured by “contact tracing”.

Thank you for pointing this out – correction made.

Lines 218-219. Consider swapping “chance” with “probability”.

Thank you for the suggestion – correction made.

Line 237 - what do you mean by “better implemented”?

We apologize for the confusing wording. We have revised this statement as follows (lines: 287-288):

“ Overall, from our sensitivity analyses, we found that TTI can be implemented more efficiently by increasing the likelihood of tracing contacts or reducing testing delays. However, TTI alone is not sufficient to control an epidemic even in a best-case scenario. ”

Line 356. I would strengthen this sentence. Delays are known to impact contact tracing effectiveness and have previously been investigated in modelling studies e.g. https://doi.org/10.1016/S2468-2667(20)30157-2

Thank you suggesting this relevant paper. We fully agree with the reviewer and we have revised the sentences as follows (line: 421-426):

“ Third, our simulations assumed an instantaneous turnaround time for contact tracing, but contact tracing often implemented through interviews conducted by an operator; thus, it may take several days to gather all information and identify contacts. Kretzschmar et al. ‘s study [69] have shown that the tracing delay has certain impact on the contact tracing effectiveness, although lower than the testing delay. Thus, the assumption of an instantaneous turnaround time for contact tracing may lead to a slightly overestimation of the effectiveness of TTI. ”

In the introduction, the authors mention that few studies of TTI have considered network structure. Please review this study by Firth et al “Using real-world network to model localised COVID-19 control strategies” . It seems relevant and should warrant a mention in the introduction and discussion. 10.1038/s41591-020-1036-8.

Thank you suggesting this relevant paper. As suggested, we have broaden our literature review to include the suggested study (lines: 109-117):

“ Several studies have attempted to incorporate temporal information in the network structure [51]. One strategy that has been adopted is the use of weighted links to indicate the frequencies or duration of contacts. For example, Firth et al. [52] constructed a static social network based on social contact data and movement data and weighted each contact by the frequency of days that a contact was observed; however, it is worth noting that this type of simplification may limit the capacity of the network model to represent various distributions of epidemic sizes [53]. A second strategy was the use of line graphs to represent the movement from source to target and the corresponding time points. This method requires specific location information and chronological order, which is highly demanding for data acquisition. ”

Worth noting in the discussion that you have used pre-pandemic contact data and it’s likely that contact rates and structures changed during the pandemic (even in the absence of government advice or mandated social restrictions). Indeed, this has been reported in many settings, and is evident through mobility data such as from Google. Further note, contact rates are most likely to spontaneously reduce when the perceived risk of infection is higher (i.e. as incidence increases). Therefore, the reactive distancing scenarios explored in your analysis need not only represent government policy scenarios, they could represent spontaneous changes in population behaviour.

We fully agree with the reviewer. We have expanded the **Discussion** to include this limitation (lines: 392-398):

“ In particular, the structure of the contact network is based on individual-level contact patterns data stratified by age and social setting. However, it is worth noting that, to reconstruct the static-temporal network, we used contact survey data collected before the COVID-19 pandemic. The pandemic has altered the contact patterns of the population in ways that go beyond the implementation of social distancing policies, including individual considerations about the perceived risk of infection [66]. Future studies are needed to estimate contact patterns in the post-pandemic era. ”

Line 365. What do you mean by “highly infectious characteristics”? High transmissibility?

We apologize for the lack of clarity of this statement. The reviewer is correct, we meant high transmissibility. The sentence has been revised as follows (lines: 437-440):

“ Our findings highlight the importance of combining TTI with an overall reduction of transmission obtained by limiting contacts regardless of their types, given the substantial proportion of asymptomatic and pre-symptomatic transmission and high reproduction number of the ancestral SARS-CoV-2 lineages as well as subsequent variants. ”

Reviewer #2 (Remarks to the Author):

I want to start by thanking the authors for the submission. Like many of us, I have read a huge amount of COVID modelling papers but this one brings novelty and methodological advancement which has been an enjoyable read.

The paper is well written and I believe methodologically sound.

We would like to thank the reviewer for taking the time to review our manuscript and for the constructive comments. We are glad that the reviewer thinks that our paper “brings novelty” and is “well written”.

Introduction
I would like to see a little bit more background on using networks in infectious diseases modelling (for example Bansal 2010: https://www.tandfonline.com/doi/ full/10.1080/17513758.2010.503376) just to highlight (what I think is) the novelty of the two layers and how the TTI system is implemented only in one layer.

Thank you for this suggestion. We have expanded the introduction adding more background on the use of networks in infectious diseases modelling. The revised parts read as follows (line: 109-117):

“ Several studies have attempted to incorporate temporal information in the network structure [51]. One strategy that has been adopted is the use of weighted links to indicate the frequencies or duration of contacts. For example, Firth et al. [52] constructed a static social network based on social contact data and movement data and weighted each contact by the frequency of days that a contact was observed; however, it is worth noting that this type of simplification may limit the capacity of the network model to represent various distributions of epidemic sizes [53]. A second strategy was the use of line graphs to represent the movement from source to target and the corresponding time points. This method requires specific location information and chronological order, which is highly demanding for data acquisition. ”

It's quite unclear what the "cost" that is mentioned and calculated several times is. I particularly note the title for Figure 3 talks about a cost but the panels of Figure 3 are all epidemiological indicators (cases, deaths, number of contacts traced). My guess is these all could have a dollar value assigned to them, but if this is the case that needs to be included in the main manuscript.

We apologize for the confusing wording we used. With “cost”, we meant the social and implementation costs associated with TTI measured in terms of peak number of individuals that are simultaneously isolated (social cost of the strategy) and the peak number of individuals that need to be traced (implementation cost of the strategy). These have been clarified in the **Methods** as follows (lines: 204-208):

“ As a measure of disease burden, we estimated the cumulative number of infections, symptomatic infections, hospitalizations, ICUs, and deaths from the simulated epidemics. As a measure of both the social and implementation cost of TTI, we estimated the peak daily number of individuals that need to be traced (i.e., those with positive test result) and the peak daily number of individuals that are simultaneously isolated. ”

Also on cost, are false positives included in the TTI scheme? From a dynamical systems perspective it doesn't matter (other than contacts being isolated when they don't need to be), but a false positive also means more contacts to be traced, and presumably more tests to be run, each of which costs money and will affect cost-benefit analysis.

We did not consider false positives in the TTI scheme. However, we fully agree with the reviewer that false positive results would lead to a larger number of traced contacts and, ultimately, a larger social and implementation cost for the strategy. This is indeed a limitation of our analysis. We have added the following paragraph to the **Discussion** to point it out (line: 427-429):

“ Fourth, we did not consider the possibility of false positive test results. Although rare, false positive results can increase the social and implementation costs of TTI. This aspect can be particularly relevant for cost-effectiveness analyses. ”

Conclusion
I'd like to see a comment saying how effective TTI can be (or that it can't fully control highly infectious conditions) in here, at the moment it reads quite generically.

Thank you for this suggestion. We have now included a specific comment about the effectiveness of TTI in the **Conclusion** (lines: 433-437):

“ Using this model, we examined the effectiveness and the social/implementation costs of TTI and identify key parameters regulating its effectiveness. We found that TTI alone could not control a COVID-19 outbreak. The infection attack rate can however be reduced by 24.5% when $R_{0}$=2.5; when NPIs are implemented to lower the transmission risk (e.g., $R_{0}$=1.3), about 48.3% of the infections can be averted. ”

Minor comments:
Line 94: However, most of modelling studies -> most modelling studies

Thank you for pointing this out – correction made.
